# Data set on the diversity and core members of bacterial community associated with two specialist fruit flies *Bactrocera melastomatos* and *B. umbrosa* (Insecta, Tephritidae)

**DOI:** 10.1016/j.dib.2022.108727

**Published:** 2022-11-08

**Authors:** Sze-Looi Song, Hoi-Sen Yong, Kah-Ooi Chua, Phaik-Eem Lim, Praphathip Eamsobhana

**Affiliations:** a Institute for Advanced Studies, Universiti Malaya, Kuala Lumpur, Malaysia; b Institute of Ocean and Earth Sciences, Universiti Malaya, Kuala Lumpur, Malaysia; c Institute of Biological Sciences, Faculty of Science, Universiti Malaya, Kuala Lumpur, Malaysia; d Centre for Research in Biotechnology for Agriculture, Universiti Malaya, Kuala Lumpur, Malaysia; e Department of Parasitology, Faculty of Medicine Siriraj Hospital, Mahidol University, Bangkok, Thailand

**Keywords:** Next generation sequencing, Miseq, Microbiome, 16S rRNA gene, Dacinae

## Abstract

*Bactrocera melastomatos* Drew & Hancock and *Bactrocera umbrosa* (Fabricius) are fruit flies of the subfamily Dacinae under the family Tephritidae [1]. *B. melastomatos* occurs in India (Andaman Island), Thailand, Peninsular Malaysia, Singapore, and Indonesia (Sumatra, Kalimantan, Java) [1] while *B. umbrosa* is distributed from southern Thailand and Malaysia to New Guinea and New Caledonia [2]. The adult male flies of *B. melastomatos* are attracted to Cue lure while the adult male flies of *B. umbrosa* are attracted to methyl eugenol [3]. Fruit flies of *Bactrocera melastomatos* infest Melastomataceae while those of *B. umbrosa* infest Moraceae. We compare the diversity of microbiota associated with the wild adult males of these two specialist fruit flies infesting different families of host plants. Targeted 16S rRNA gene (V3-V4 region) was sequenced using the Illumina MiSeq platform. Six bacterial phyla (*Actinobacteria, Armatimonadetes, Bacteroidetes, Cyanobacteria*/*Melainabacteria* group, *Firmicutes, Proteobacteria*) were detected at 97% similarity clustering and 0.001% abundance filtering. Four phyla (*Actinobacteria, Bacteroidetes, Firmicutes, Proteobacteria*) were present in all the specimens studied. *Proteobacteria* was the predominant phylum in both *B. melastomatos* and *B. umbrosa. Enterobacteriaceae* was the predominant family in UM *B. melastomatos* and *B. umbrosa*, and Orbaceae was the predominant family in Awana *B. melastomatos. Klebsiella* was the predominant genus in *B. umbrosa, Citrobacter* in UM *B. melastomatos*, and *Orbus* in Awana *B. melastomatos*. Double *Wolbachia* infections were present in UM *B. melastomatos*. In general, the bacterial diversity and richness varied within and between the samples of *B. melastomatos* and *B. umbrosa*.

**Specifications Table**

**Table:** 

Subject	Microbiology: Microbiome
Specific subject area	Metagenomics
Type of data	Tables Figures Fastq files
How the data were acquired	Illumina MiSeq System (2 × 250 bp paired-end reads)
Data format	Raw and Analyzed
Description of data collection	Wild adult male flies of *B. melastomatos* were collected by means of Cue lure, while those of *B. umbrosa* were collected by methyl eugenol. These fruit flies were collected in Peninsular Malaysia – *B. melastomatos*: 2 specimens from Universiti Malaya (UM) campus and 3 from Awana Genting Resort (Awana); *B. umbrosa*: 5 specimens from Universiti Malaya campus. Total DNA was extracted from the sample and the 16S rRNA gene amplicon (V3-V4 region) was sequenced by the Illumina MiSeq system.
Data source location	Institution: Universiti Malaya City/Town/Region: Kuala Lumpur Country: Malaysia Latitude and longitude (and GPS coordinates, if possible) for collected samples/data: Universiti Malaya (UM) campus (3.1201^o^N, 101.6544^o^E) and Awana Genting Resort (Awana) (3.2381^o^N, 101.4680^o^E)
Data accessibility	Repository name: GenBank Sequence Read Archive [4] Data identification number: Data are available at the NCBI with Bioproject PRJNA528573 Direct URL to data: https://www.ncbi.nlm.nih.gov/Traces/study/?acc=PRJNA528573&o=acc_s%3Aa

## Value of the Data

• The data provide information on the core members and different taxa of the bacterial community associated with *B. melastomatos* which infests only the fruits of Melastomataceae and *B. umbrosa* which infests only Artocarpus fruits of Moraceae.
• The data are useful for comparative analysis of abundance and core members of the bacterial community with other specialist as well as generalist fruit flies.
• The data are useful for culture-dependent technique on the microbiota associated with these two species of specialist fruit flies.
• The data are valuable for developing pest management programme in controlling the fruit flies infesting host plants.

## 1 Data Description

**Fig. 1:**
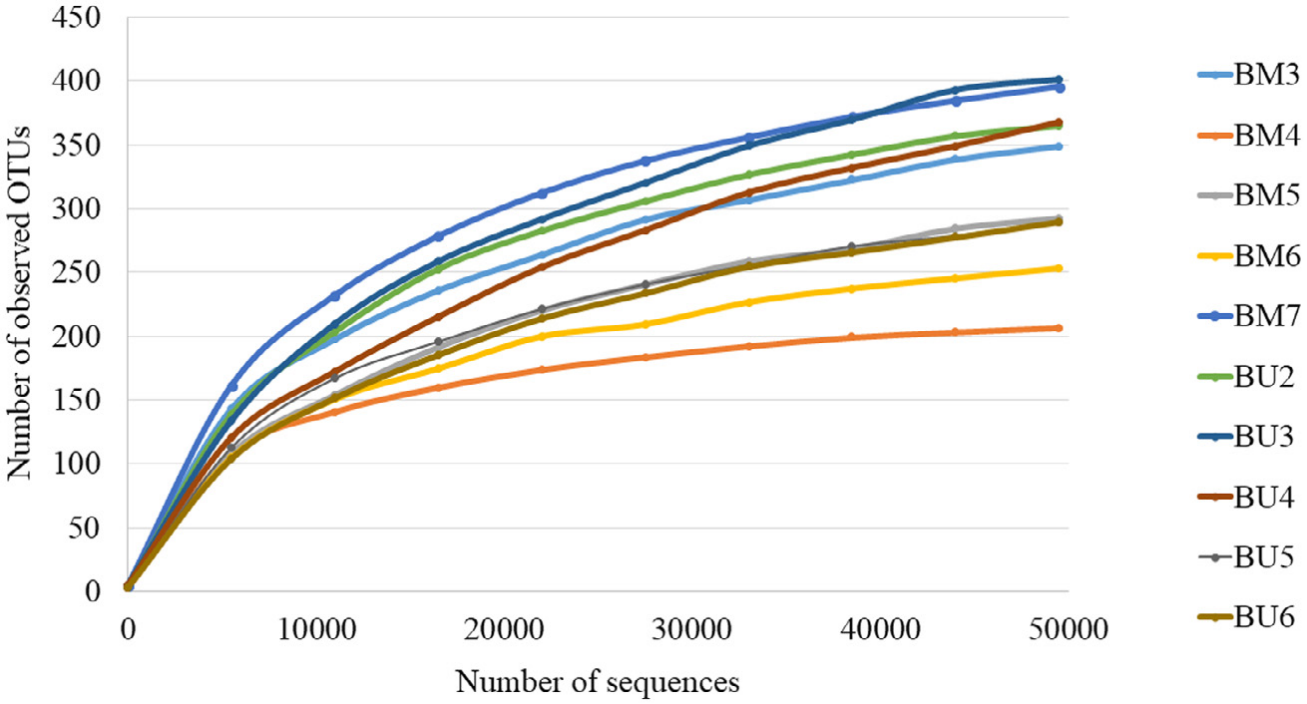
The richness of bacterial communities in *Bactrocera melastomatos* (BM3–BM5, Awana Genting Resort; BM6–BM7, Universiti Malaya) and *Bactrocera umbrosa* samples (BU2–BU6, Universiti Malaya) from Peninsular Malaysia.

The high throughput sequencing generated a total of 2205662 raw sequence reads. After quality filtering and chimera removal, the samples were obtained with sequences ranging from about 59907 in BU2 to 85783 in BM7. The number of reads varied among the specimens of *B. melastomatos* (74176–76713 in Awana samples and 61018–85783 in UM samples) and *B. umbrosa* (59907–81575). The species richness varied considerably within and across the three groups of samples (Fig. 1). The raw datasets for 16S rRNA gene amplicon sequencing generated for this paper have been deposited in the GenBank Sequence Read Archive (accession number PRJNA528573).

The overall bacterial community in the samples of *B. melastomatos* and *B. umbrosa* consisted of six phyla, 11 classes, 23 orders, 30 families, 64 genera, and 122 putative species (Table 1; Supplementary Tables S1, S2). Of the six bacterial phyla, four – *Actinobacteria, Bacteroidetes, Firmicutes*, and *Proteobacteria* – were represented in all the fruit fly specimens, forming the core members of the bacterial community (Table 1; Supplementary Tables S1–S3). *Proteobacteria* was the predominant phylum (relative abundance from 78.84 ± 22.71% in *B. umbrosa* to 90.25 ± 6.99% in Awana *B. melastomatos*), followed by *Bacteroidetes* (5.18 ± 0.51% in UM *B. melastomatos* to 8.54 ± 7.66% in Awana *B. melastomatos*), *Firmicutes* (from 0.87 ± 0.74% in Awana *B. melastomatos* to 17.09 ± 15.60% in *B. umbrosa*), and *Actinobacteria* (the least abundant with very low relative abundance, from 0.01 ± 0.01% in UM *B. melastomatos* to 0.05 ± 0.06% in *B. umbrosa*) (Supplementary Table S3).

**Table 1:** The relative abundance (%) of the bacterial phylum OTUs in the field-caught adult male flies of *Bactrocera melastomatos* (BM3-BM5, Awana Genting Resort; BM6-BM7, Universiti Malaya) and *Bactrocera umbrosa* (BU2-BU6, Universiti Malaya) determined by 16S rRNA gene sequencing.

Phylum	BM3	BM4	BM5	BM6	BM7	BU2	BU3	BU4	BU5	BU6
*Actinobacteria*	0.10	0.01	0.01	0.00	0.01	0.16	0.03	0.01	0.06	0.00
*Armatimonadetes*	0	0.00	0.01	0	0	0	0	0	0	0
*Bacteroidetes*	3.72	17.37	4.52	5.54	4.82	1.93	27.17	3.80	0.16	6.83
*Cyanobacteria*/										
*Melainabacteria* group	0.03	0.01	0.13	0	0.00	0.00	0.01	0.00	0.00	0.00
*Firmicutes*	1.26	0.01	1.35	31.63	0.01	4.02	15.52	35.32	0.11	30.48
*Proteobacteria*	94.65	82.19	93.91	62.78	94.90	93.94	57.03	60.80	99.31	62.44
Unassigned	0.25	0.40	0.08	0.05	0.26	0.36	0.24	0.06	0.36	0.24

Of the four core phyla, the respective number of core OTUs were: *Proteobacteria* – 4 classes, 4 orders, 4 families, 9 genera, 11 species; *Bacteroidetes* – 2 classes, 2 orders, 2 families, 1 genus, 1 species; *Firmicutes* – 1 class, 1 order, 1 family, 1 genus, 1 species; *Actinobacteria* – 1 class (Table 2, Supplementay Table S1). *Gammaproteobacteria* was the predominant class (mean relative abundance of 80.52 ± 1.06% in Awana *B. melastomatos*, 59.94 ± 36.28% in UM *B. melastomatos* and 71.79 ± 21.77% in *B. umbrosa*). In Awana *B. melastomatos*, the predominant OTUs were: order Orbales (47.87 ± 41.37%), family Orbaceae (47.87 ± 41.37%), genus *Orbus* (45.59 ± 39.35%), and species *Orbus sasakiae* (45.59 ± 39.34%) (Supplementary Tables S1, 3). On the other hand, the predominant OTUs in UM *B. melastomatos* were: order *Enterobacteriales* (43.17 ± 59.95%), family *Enterobacteriaceae* (43.17 ± 59.95%), genus *Citrobacter* (23.26 ± 32.55%), and species *Citrobacter freundii* (23.19 ± 32.45%). In *B. umbrosa*, the predominant OTUs were: order *Enterobacteriales* (56.03 ± 41.13%), family *Enterobacteriaceae* (56.03 ± 41.13%), genus *Klebsiella* (26.53 ± 35.06%), and species *Klebsiella oxytoca* (25.75 ± 34.75%).

**Table 2:** The core bacterial OTUs detected in the samples of *Bactrocera melastomatos* and *Bactrocera umbrosa* from Peninsular Malaysia.

Phylum	Class	Order	Family	Genus	Species
*Actinobacteria*	*Actinobacteria*				
*Bacteroidetes*	*Bacteroidia* *Flavobacteria*	*Bacteroidales* *Flavobacteriales*	*Porphyromonadaceae* *Flavobacteriaceae*	*Microbacter*	*M. margulisiae*
*Firmicutes*	*Bacilli*	*Lactobacillales*	*Enterococcaceae*	*Enterococcus*	*E. moraviensis*
*Proteobacteria*	*Alphaproteobacteria* *Betaproteoacteria* *Deltaproteobacteria* *Gammaproteobacteria*	*Burkholderiales* *Desulfovibrinales* *Enterobacteriales* *Orbales*	*Burkholdriaceae* *Desulfovibrionaceae* *Enterobacteriaceae* *Orbaceae*	*Burkholderia* *Desulfovibrio* *Citrobacter* *Enterobacter* *Erwinia* *Klebsiella* *Raoultella* *Siccibacter* *Orbus*	*B. cepacia* *D. cuneatus* *C. freundii* *E. aerogenes* *E. cloacae* *K. oxytoca* *K. pneumoniae* *K. quasipneumoniae* *R. planticola* *S. turicensis* *O. sasakiae*
Total 4	8	7	7	11	13

**Fig. 2:**
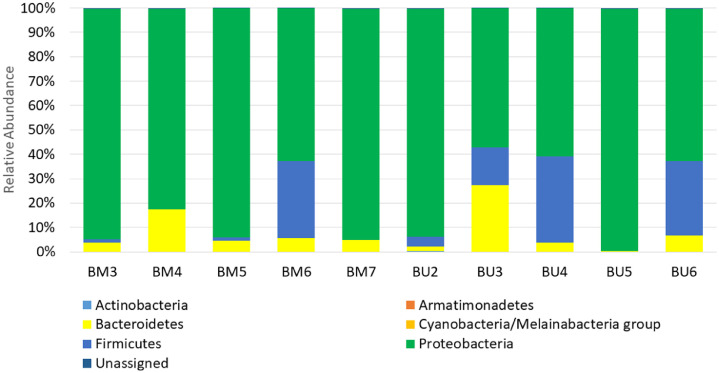
The relative abundance (%) of bacterial phyla in samples of wild adult male *Bactrocera melastomatos* (BM) and *Bactrocera umbrosa* (BU) from Peninsular Malaysia; BM3–BM5 (Awana Genting Resort), BM6–BM7 (Universiti Malaya), BU2–BU6 (Universiti Malaya).

**Fig. 3:**
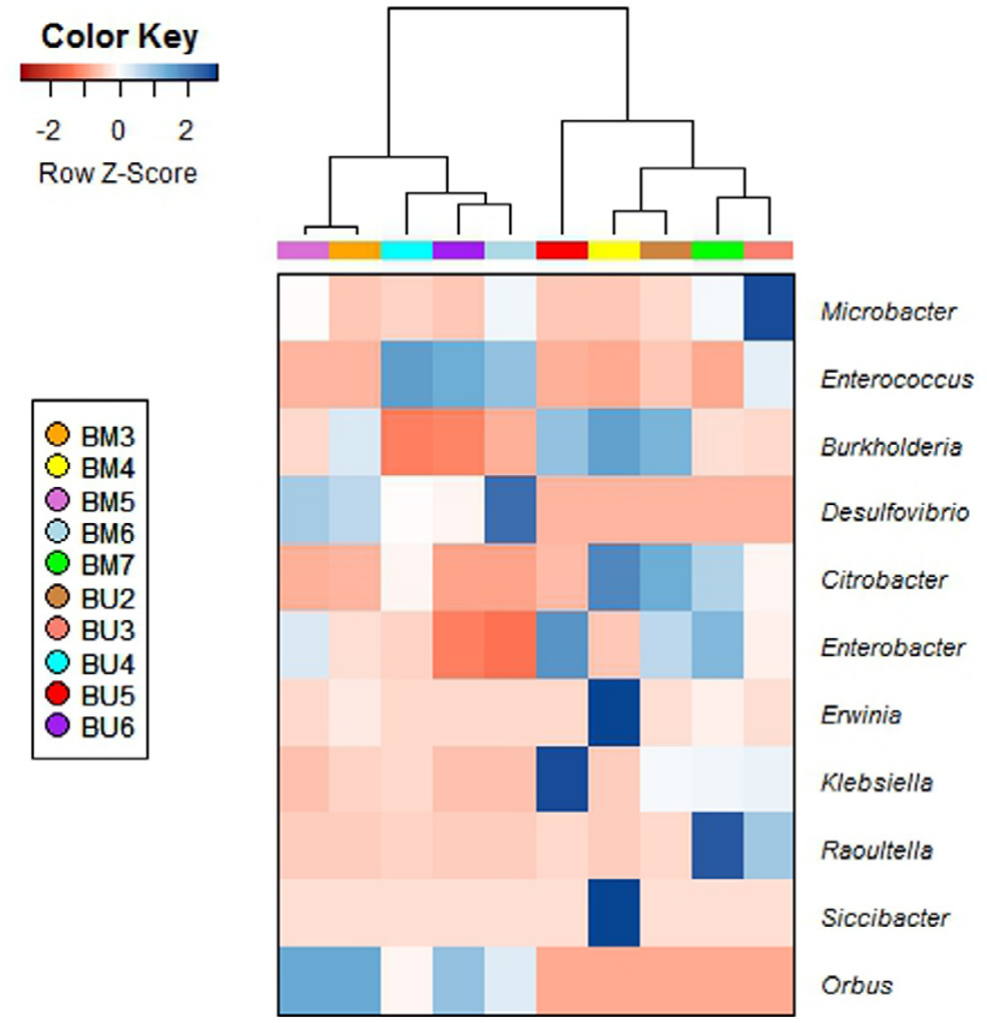
The frequency distribution of 11 core bacterial genera in samples of wild adult male *Bactrocera melastomatos* (BM) and *Bactrocera umbrosa* (BU) from Peninsular Malaysia; BM3–BM5 (Awana Genting Resort), BM6–BM7 (Universiti Malaya), BU2–BU6 (Universiti Malaya).

**Fig. 4:**
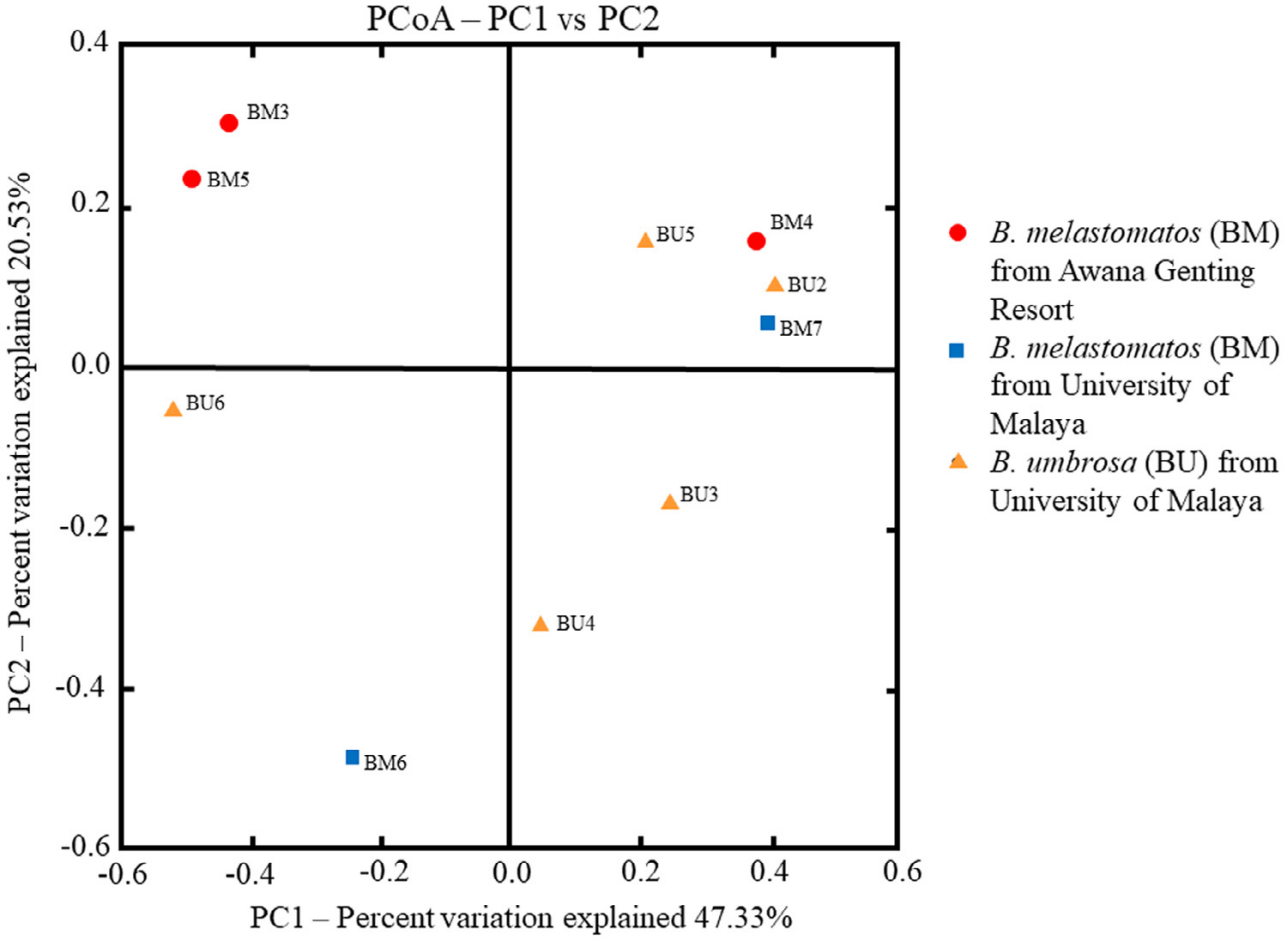
The comparison of bacterial genera diversity in samples of wild adult male *Bactrocera melastomatos* (BM) and *Bactrocera umbrosa* (BU) from Peninsular Malaysia; BM3–BM5 (Awana Genting Resort), BM6–BM7 (Universiti Malaya), BU2–BU6 (Universiti Malaya).

In general, the bacterial OTU diversity varied within and between the samples of *B. melastomatos* and *B. umbrosa* (Table 3; Fig. 2, Fig. 3, Fig. 4). The richness also varied within and between the samples. The bacterial community in the UM *B. melastomatos* samples were more diverse than the Awana *B. melastomatos* and *B. umbrosa* samples. On the other hand, the bacterial community in the *B. umbrosa* samples were more variable. Non-parametric statistical test analysis of similarity (ANOSIM) showed non significant differences in bacterial diversity between the samples (*p* = 0.30; *R* = 0.083; number of permutations = 999). Both the Shannon index and Simpson index indicate that the mean bacterial diversity (Awana *B. melastomatos* sample: H = 1.96±0.22, D = 0.50±0.04; UM *B. melastomatos* sample: H = 2.75±0.07, D = 0.75±0.03; *B. umbrosa* sample: H = 2.14±0.30, D = 0.59±0.10) was not significantly different between the samples – ANOVA test: Shannon index *F* = 1.29, *p* = 0.33; Simpson index *F* = 1.33, *p* = 0.32.

**Table 3:** The diversity of bacterial OTUs in the samples of *Bactrocera melastomatos* (BM) and *Bactrocera umbrosa* (BU) from Peninsular Malaysia; BM3–BM5, Awana Genting Resort; BM6–BM7, Universiti Malaya; BU2–BU6, Universiti Malaya; PD, phylogenetic diversity.

Sample	Shannon (H)	Simpson (D)	Goods coverage	Chao1	Observed OTUs	PD whole tree
BM3	2.32	0.55	1.00	450.85	365	13.05
BM4	1.57	0.43	1.00	253.16	210	7.29
BM5	1.99	0.51	1.00	400.02	305	12.06
BM6	2.81	0.78	1.00	291.50	262	11.37
BM7	2.68	0.72	1.00	464.52	406	10.33
BU2	1.93	0.50	1.00	438.02	375	11.57
BU3	2.79	0.80	1.00	520.02	419	12.89
BU4	2.75	0.77	1.00	507.61	387	13.29
BU5	1.14	0.26	1.00	350.11	301	8.97
BU6	2.07	0.62	1.00	355.02	299	13.15

## 2 Experimental Design, Materials and Methods

### 2.1 Materials and Methods

#### 2.1.1 Sample Collection and DNA Extraction

Wild adult male flies of *B. melastomatos* were collected by means of Cue lure, while those of *B. umbrosa* were collected by methyl eugenol. These fruit flies were collected in Peninsular Malaysia – *B. melastomatos*: 2 specimens from Universiti Malaya (UM) campus (3.1201^o^N, 101.6544^o^E) and 3 from Awana Genting Resort (Awana) (3.2381^o^N, 101.4680^o^E); *B. umbrosa*: 5 specimens from Universiti Malaya campus. They were preserved immediately in absolute ethanol and stored in deep freezer until used for DNA extraction. These fruit flies are not endangered or protected by law and permits are not required to study them. The entire single insect was used for DNA extraction following the manufacturer's instructions for G-spin^TM^ Total DNA Extraction Mini Kit (iNtRON Biotechnology, Inc, Korea).

#### 2.1.2 Targeted Metagenomics Sequencing

Demultiplexed raw sequences were extracted from the Illumina MiSeq system in FASTQ format and FastQC software was used to evaluate the quality of sequences [5]. The CLC Genomic Workbench v.7.5.1 was used to pair, merge, trim and filter the raw sequences (https://www.qiagenbioinformatics.com/). Ambiguous bases, low quality reads and sequences with read length below 200 bp were discarded. UCHIME was used to identify and remove the potential chimeric sequences [6,7]. UCLUST by open-reference OTU picking approach in Quantitative Insights into Microbial Ecology (Qiime v.1.9.0) was used to cluster the sequence reads into Operational Taxonomic Units (OTUs) at 97% similarity [6,8]. A representative sequence for each OTU was selected for taxonomic assignment with reference to the Greengene 13_8-release database [9] and additionally blasted against the NCBI 16S microbial database to gain additional insight into species level.

#### 2.1.3 Bioinformatics and Statistical Analyses

Alpha and beta diversity analyses, and Principal Coordinate Analysis (PCoA) were performed as earlier described [10,11]. One-way ANOVA with post-hoc Tukey HSD test was used to compare the mean relative abundance of OTUs of different samples. A heatmap with OTU abundance and hierarchical clustering of samples was generated using R version 3.2.4 with Euclidean distances specified [12].

## Ethics Statements

These fruit flies are not endangered or protected by law and permits are not required to study them.

## CRediT Author Statement

**S.-L. Song:** Writing, Methodology, Software; **H.-S. Yong:** Writing – original draft preparation, Supervision, Conceptualization; **K.-O. Chua:** Writing, Methodology; **P.-E. Lim:** Writing – review & editing; **P. Eamsobhana:** Writing – review & editing.

## Declaration of Competing Interest

The authors declare that they have no known competing financial interests or personal relationships that could have appeared to influence the work reported in this paper.

The authors declare the following financial interests/personal relationships which may be considered as potential competing interests.

## Data Availability

Bioproject PRJNA528573 (Original data) (GenBank Sequence Read Archive).

## Appendix Supplementary materials

Image, application 1
