# Supplementary material for: Data set on the diversity and core members of bacterial community associated with two specialist fruit flies Bactrocera melastomatos and B. umbrosa (Insecta, Tephritidae)
Source: Data Brief. 2022 Nov 8;45:108727. doi: 10.1016/j.dib.2022.108727 (PMC9679684; doi:10.1016/j.dib.2022.108727)
Supplement: Supplementary file 1 [file mmc1.docx]

**Data Set on The Diversity and Core Members of Bacterial Community Associated with Two Specialist Fruit Flies *Bactrocera melastomatos* and *B. umbrosa* (Insecta, Tephritidae)**

**Sze-Looi Song^1,2*^, Hoi-Sen Yong^3^, Kah-Ooi Chua^4^**^*^**, Phaik-Eem Lim^2^, Praphathip Eamsobhana^5^**

^1^Institute for Advanced Studies, Universiti Malaya, Kuala Lumpur, Malaysia

^2^Institute of Ocean and Earth Sciences, Universiti Malaya, Kuala Lumpur, Malaysia

^3^Institute of Biological Sciences, Faculty of Science, Universiti Malaya, Kuala Lumpur, Malaysia

^4^Centre for Research in Biotechnology for Agriculture, Universiti Malaya, Kuala Lumpur, Malaysia

^5^Department of Parasitology, Faculty of Medicine Siriraj Hospital, Mahidol University, Bangkok, Thailand

*Corresponding author

E-mail address: szelooi@um.edu.my (S.-L. Song)

E-mail address: kahooi@um.edu.my (K.-O. Chua)

**Supplementary Table S1** Relative abundance (filtered at 0.001%) of bacterial OTUs in *Bactrocera melastomatos* (BM) caught in Awana Genting Resort and Universiti Malaya and *Bactrocera umbrosa* (BU) caught in Universiti Malaya. BM3–BM5, Awana Genting Resort; BM6–BM7, Universiti Malaya; BU2–BU6, Universiti Malaya.

**Phylum**

| Phylum | BM3 | BM4 | BM5 | BM6 | BM7 | BU2 | BU3 | BU4 | BU5 | BU6 |
| --- | --- | --- | --- | --- | --- | --- | --- | --- | --- | --- |
| Actinobacteria | 0.10 | 0.01 | 0.01 | 0.00 | 0.01 | 0.16 | 0.03 | 0.01 | 0.06 | 0.00 |
| Armatimonadetes | 0 | 0.00 | 0.01 | 0 | 0 | 0 | 0 | 0 | 0 | 0 |
| Bacteroidetes | 3.72 | 17.37 | 4.52 | 5.54 | 4.82 | 1.93 | 27.17 | 3.80 | 0.16 | 6.83 |
| Cyanobacteria/Melainabacteria group | 0.03 | 0.01 | 0.13 | 0 | 0.00 | 0.00 | 0.01 | 0.00 | 0.00 | 0.00 |
| Firmicutes | 1.26 | 0.01 | 1.35 | 31.63 | 0.01 | 4.02 | 15.52 | 35.32 | 0.11 | 30.48 |
| Proteobacteria | 94.65 | 82.19 | 93.91 | 62.78 | 94.90 | 93.54 | 57.03 | 60.80 | 99.31 | 62.44 |
| Unassigned | 0.25 | 0.40 | 0.08 | 0.05 | 0.26 | 0.36 | 0.24 | 0.06 | 0.36 | 0.24 |

**Class**

|  | BM3 | BM4 | BM5 | BM6 | BM7 | BU2 | BU3 | BU4 | BU5 | BU6 |
| --- | --- | --- | --- | --- | --- | --- | --- | --- | --- | --- |
| Actinobacteria; Actinobacteria | 0.10 | 0.01 | 0.01 | 0.00 | 0.01 | 0.16 | 0.03 | 0.01 | 0.06 | 0.00 |
| Armatimonadetes; Chthonomonadetes | 0 | 0.00 | 0.01 | 0 | 0 | 0 | 0 | 0 | 0 | 0 |
| Bacteroidetes; Bacteroidia | 3.72 | 0.01 | 4.44 | 5.38 | 4.80 | 1.60 | 27.07 | 1.04 | 0.07 | 6.67 |
| Bacteroidetes; Flavobacteriia | 0.00 | 17.36 | 0.08 | 0.16 | 0.02 | 0.33 | 0.10 | 2.76 | 0.09 | 0.16 |
| Cyanobacteria/Melainabacteria group; Cyanobacteria | 0.03 | 0.01 | 0.13 | 0 | 0.00 | 0.00 | 0.01 | 0.00 | 0.00 | 0.00 |
| Firmicutes; Bacilli | 1.26 | 0.01 | 1.34 | 31.63 | 0.01 | 4.02 | 15.52 | 35.32 | 0.11 | 30.48 |
| Firmicutes; Tissierellia | 0 | 0 | 0.00 | 0.00 | 0 | 0 | 0 | 0 | 0.00 | 0 |
| Proteobacteria; Alphaproteobacteria | 1.17 | 0.01 | 0.00 | 3.33 | 8.91 | 0.04 | 0.02 | 0.01 | 0.02 | 0.01 |
| Proteobacteria; Betaproteobacteria | 0.61 | 0.83 | 0.40 | 0.27 | 0.39 | 0.91 | 0.42 | 0.17 | 0.84 | 0.17 |
| Proteobacteria; Deltaproteobacteria | 11.96 | 0.01 | 14.18 | 24.89 | 0.01 | 0.01 | 0.02 | 6.05 | 0.01 | 5.46 |
| Proteobacteria; Gammaproteobacteria | 80.90 | 81.34 | 79.32 | 34.29 | 85.59 | 92.58 | 56.56 | 54.57 | 98.45 | 56.80 |
| Unassigned; Unassigned | 0.25 | 0.40 | 0.08 | 0.05 | 0.26 | 0.36 | 0.24 | 0.06 | 0.36 | 0.24 |

**Order**

|  | BM3 | BM4 | BM5 | BM6 | BM7 | BU2 | BU3 | BU4 | BU5 | BU6 |
| --- | --- | --- | --- | --- | --- | --- | --- | --- | --- | --- |
| Actinobacteria; Corynebacteriales | 0.09 | 0 | 0 | 0 | 0 | 0.01 | 0.00 | 0 | 0.00 | 0 |
| Actinobacteria; Micrococcales | 0.00 | 0.01 | 0 | 0.00 | 0.01 | 0.00 | 0.01 | 0.01 | 0.00 | 0 |
| Actinobacteria; Propionibacteriales | 0.01 | 0.01 | 0.01 | 0 | 0.00 | 0.15 | 0.02 | 0.00 | 0.05 | 0.00 |
| Chthonomonadetes; Chthonomonadales | 0 | 0.00 | 0.01 | 0 | 0 | 0 | 0 | 0 | 0 | 0 |
| Bacteroidia; Bacteroidales | 3.72 | 0.01 | 4.44 | 5.38 | 4.80 | 1.60 | 27.07 | 1.04 | 0.07 | 6.67 |
| Flavobacteriia; Flavobacteriales | 0.00 | 17.36 | 0.08 | 0.16 | 0.02 | 0.33 | 0.10 | 2.76 | 0.09 | 0.16 |
| Cyanobacteria; Stigonematales | 0.03 | 0.01 | 0.13 | 0 | 0.00 | 0.00 | 0.01 | 0.00 | 0.00 | 0.00 |
| Bacilli; Lactobacillales | 1.26 | 0.01 | 1.34 | 31.63 | 0.01 | 4.02 | 15.52 | 35.32 | 0.11 | 30.48 |
| Tissierellia; Tissierellales | 0 | 0 | 0.00 | 0.00 | 0 | 0 | 0 | 0 | 0.00 | 0 |
| Alphaproteobacteria; Caulobacterales | 0.00 | 0 | 0 | 0 | 0.00 | 0.01 | 0 | 0 | 0.00 | 0 |
| Alphaproteobacteria; Rhizobiales | 0.01 | 0.00 | 0.00 | 0 | 0.01 | 0.01 | 0 | 0.00 | 0.00 | 0 |
| Alphaproteobacteria; Rhodospirillales | 1.15 | 0.00 | 0 | 0 | 0 | 0.02 | 0.02 | 0.01 | 0.01 | 0.01 |
| Alphaproteobacteria; Rickettsiales | 0 | 0 | 0.00 | 3.33 | 8.89 | 0.00 | 0.00 | 0 | 0 | 0 |
| Alphaproteobacteria; Sphingomonadales | 0 | 0.01 | 0 | 0 | 0 | 0.00 | 0 | 0 | 0.00 | 0 |
| Betaproteobacteria; Burkholderiales | 0.61 | 0.83 | 0.40 | 0.27 | 0.39 | 0.91 | 0.42 | 0.17 | 0.84 | 0.17 |
| Deltaproteobacteria; Desulfovibrionales | 11.96 | 0.01 | 14.18 | 24.89 | 0.01 | 0.01 | 0.02 | 6.05 | 0.01 | 5.46 |
| Gammaproteobacteria; Aeromonadales | 0.01 | 0 | 0.01 | 0 | 0.01 | 0 | 0 | 0 | 0 | 0 |
| Gammaproteobacteria; Chromatiales | 0.01 | 0 | 0.02 | 0.04 | 0 | 0 | 0 | 0.00 | 0 | 0.00 |
| Gammaproteobacteria; Enterobacteriales | 11.49 | 81.15 | 5.21 | 0.78 | 85.56 | 92.53 | 56.49 | 31.96 | 98.42 | 0.73 |
| Gammaproteobacteria; Oceanospirillales | 0 | 0.00 | 0.00 | 0 | 0.01 | 0.03 | 0.01 | 0 | 0.01 | 0.00 |
| Gammaproteobacteria; Orbales | 69.36 | 0.18 | 74.08 | 33.47 | 0.01 | 0.01 | 0.04 | 22.60 | 0.02 | 56.05 |
| Gammaproteobacteria; Pseudomonadales | 0.03 | 0 | 0.00 | 0 | 0 | 0.01 | 0.02 | 0.01 | 0 | 0.01 |
| Gammaproteobacteria; Thiotrichales | 0.01 | 0 | 0 | 0 | 0 | 0.00 | 0 | 0 | 0 | 0 |
| Unassigned | 0.25 | 0.40 | 0.08 | 0.05 | 0.26 | 0.36 | 0.24 | 0.06 | 0.36 | 0.24 |

**Family**

|  | BM3 | BM4 | BM5 | BM6 | BM7 | BU2 | BU3 | BU4 | BU5 | BU6 |
| --- | --- | --- | --- | --- | --- | --- | --- | --- | --- | --- |
| Corynebacteriales; Corynebacteriaceae | 0 | 0 | 0 | 0 | 0 | 0.01 | 0.00 | 0 | 0.00 | 0 |
| Corynebacteriales; Mycobacteriaceae | 0.08 | 0 | 0 | 0 | 0 | 0 | 0 | 0 | 0 | 0 |
| Corynebacteriales; Williamsiaceae | 0.01 | 0 | 0 | 0 | 0 | 0 | 0 | 0 | 0 | 0 |
| Micrococcales; Microbacteriaceae | 0.00 | 0.01 | 0 | 0.00 | 0.01 | 0.00 | 0.01 | 0.01 | 0.00 | 0 |
| Propionibacteriales; Propionibacteriaceae | 0.01 | 0.01 | 0.01 | 0 | 0.00 | 0.15 | 0.02 | 0.00 | 0.05 | 0.00 |
| Chthonomonadales; Chthonomonadaceae | 0 | 0.00 | 0.01 | 0 | 0 | 0 | 0 | 0 | 0 | 0 |
| Bacteroidales; Bacteroidaceae | 0 | 0 | 0.06 | 0.07 | 0.07 | 0.02 | 0.25 | 0.02 | 0.00 | 0 |
| Bacteroidales; Porphyromonadaceae | 3.72 | 0.01 | 4.38 | 5.31 | 4.73 | 1.57 | 26.81 | 1.02 | 0.07 | 6.67 |
| Flavobacteriales; Flavobacteriaceae | 0.00 | 17.36 | 0.08 | 0.16 | 0.02 | 0.33 | 0.10 | 2.76 | 0.09 | 0.16 |
| Stigonematales; Hapalosiphonaceae | 0.03 | 0.01 | 0.13 | 0 | 0.00 | 0.00 | 0.01 | 0.00 | 0.00 | 0.00 |
| Lactobacillales; Enterococcaceae | 1.18 | 0.01 | 1.34 | 25.07 | 0.01 | 3.94 | 15.49 | 34.96 | 0.06 | 29.51 |
| Lactobacillales; Leuconostocaceae | 0 | 0 | 0 | 0 | 0 | 0 | 0 | 0 | 0 | 0.01 |
| Lactobacillales; Streptococcaceae | 0.07 | 0 | 0 | 6.56 | 0 | 0.08 | 0.03 | 0.36 | 0.05 | 0.96 |
| Tissierellales; Peptoniphilaceae | 0 | 0 | 0.00 | 0.00 | 0 | 0 | 0 | 0 | 0.00 | 0 |
| Caulobacterales; Caulobacteraceae | 0.01 | 0 | 0.01 | 0 | 0.01 | 0 | 0 | 0 | 0 | 0 |
| Rhizobiales; Bradyrhizobiaceae | 0.61 | 0.83 | 0.40 | 0.27 | 0.39 | 0.91 | 0.42 | 0.17 | 0.84 | 0.17 |
| Rhizobiales; Methylobacteriaceae | 0.00 | 0 | 0 | 0 | 0.00 | 0.01 | 0 | 0 | 0.00 | 0 |
| Rhodospirillales; Acetobacteraceae | 0.00 | 0 | 0.02 | 0.01 | 0 | 0 | 0 | 0 | 0 | 0 |
| Rickettsiales; Anaplasmataceae | 0.01 | 0 | 0 | 0.03 | 0 | 0 | 0 | 0.00 | 0 | 0.00 |
| Sphingomonadales; Sphingomonadaceae | 11.96 | 0.01 | 14.18 | 24.89 | 0.01 | 0.01 | 0.02 | 6.05 | 0.01 | 5.46 |
| Burkholderiales; Burkholderiaceae | 11.49 | 81.15 | 5.21 | 0.78 | 85.56 | 92.53 | 56.49 | 31.96 | 98.42 | 0.73 |
| Desulfovibrionales; Desulfovibrionaceae | 0 | 0.00 | 0.00 | 0 | 0.01 | 0.03 | 0.01 | 0 | 0.01 | 0.00 |
| Aeromonadales; Aeromonadaceae | 69.36 | 0.18 | 74.08 | 33.47 | 0.01 | 0.01 | 0.04 | 22.60 | 0.02 | 56.05 |
| Chromatiales; Chromatiaceae | 0.03 | 0 | 0.00 | 0 | 0 | 0.01 | 0.02 | 0.01 | 0 | 0.01 |
| Chromatiales; Granulosicoccaceae | 0.00 | 0.00 | 0.00 | 0 | 0.01 | 0.01 | 0 | 0 | 0.00 | 0 |
| Enterobacteriales; Enterobacteriaceae | 0.01 | 0 | 0 | 0 | 0 | 0 | 0 | 0.00 | 0 | 0 |
| Oceanospirillales; Halomonadaceae | 1.15 | 0.00 | 0 | 0 | 0 | 0.02 | 0.02 | 0.01 | 0.01 | 0.01 |
| Orbales; Orbaceae | 0 | 0 | 0.00 | 3.33 | 8.89 | 0.00 | 0.00 | 0 | 0 | 0 |
| Pseudomonadales; Pseudomonadaceae | 0 | 0.01 | 0 | 0 | 0 | 0.00 | 0 | 0 | 0.00 | 0 |
| Thiotrichales; Thiotrichaceae | 0.01 | 0 | 0 | 0 | 0 | 0.00 | 0 | 0 | 0 | 0 |
| Unassigned | 0.25 | 0.40 | 0.08 | 0.05 | 0.26 | 0.36 | 0.24 | 0.06 | 0.36 | 0.24 |

**Genus**

|  | BM3 | BM4 | BM5 | BM6 | BM7 | BU2 | BU3 | BU4 | BU5 | BU6 |
| --- | --- | --- | --- | --- | --- | --- | --- | --- | --- | --- |
| Corynebacteriaceae; *Corynebacterium* | 0 | 0 | 0 | 0 | 0 | 0.01 | 0.00 | 0 | 0.00 | 0 |
| Mycobacteriaceae; *Mycobacterium* | 0.00 | 0.01 | 0 | 0.00 | 0.01 | 0.00 | 0.01 | 0.01 | 0.00 | 0 |
| Williamsiaceae; *Williamsia* | 0.08 | 0 | 0 | 0 | 0 | 0 | 0 | 0 | 0 | 0 |
| Microbacteriaceae; *Leifsonia* | 0.01 | 0.01 | 0.01 | 0 | 0.00 | 0.15 | 0.02 | 0.00 | 0.05 | 0.00 |
| Propionibacteriaceae; *Propionibacterium* | 0.01 | 0 | 0 | 0 | 0 | 0 | 0 | 0 | 0 | 0 |
| Chthonomonadaceae; *Chthonomonas* | 0 | 0.00 | 0.01 | 0 | 0 | 0 | 0 | 0 | 0 | 0 |
| Bacteroidaceae; *Bacteroides* | 0 | 0 | 0.06 | 0.07 | 0.07 | 0.02 | 0.25 | 0.02 | 0.00 | 0 |
| Porphyromonadaceae; *Dysgonomonas* | 0.00 | 0 | 0 | 0.15 | 0 | 0.32 | 0.03 | 2.75 | 0.08 | 0.16 |
| Porphyromonadaceae; *Microbacter* | 0 | 17.36 | 0 | 0 | 0 | 0 | 0 | 0 | 0 | 0 |
| Porphyromonadaceae; *Parabacteroides* | 0 | 0 | 0.08 | 0.02 | 0.02 | 0.01 | 0.07 | 0.01 | 0.00 | 0 |
| Porphyromonadaceae; *Porphyromonas* | 3.72 | 0 | 0.00 | 0.02 | 0 | 0 | 0 | 0.00 | 0.00 | 6.67 |
| Flavobacteriaceae; *Chishuiella* | 0.00 | 0.01 | 4.25 | 5.26 | 4.69 | 1.55 | 26.68 | 1.01 | 0.07 | 0.00 |
| Flavobacteriaceae; *Chryseobacterium* | 0 | 0 | 0.02 | 0.00 | 0 | 0 | 0 | 0 | 0 | 0 |
| Flavobacteriaceae; *Flavobacterium* | 0 | 0 | 0.10 | 0.02 | 0.03 | 0.02 | 0.13 | 0.00 | 0.00 | 0 |
| Hapalosiphonaceae; *Mastigocoleus* | 0.03 | 0.01 | 0.13 | 0 | 0.00 | 0.00 | 0.01 | 0.00 | 0.00 | 0.00 |
| Enterococcaceae; *Enterococcus* | 1.18 | 0.01 | 1.32 | 24.99 | 0.01 | 3.93 | 15.46 | 34.93 | 0.06 | 29.45 |
| Enterococcaceae; *Vagococcus* | 0.01 | 0.00 | 0.02 | 0.08 | 0 | 0.01 | 0.03 | 0.03 | 0 | 0.06 |
| Leuconostocaceae; *Leuconostoc* | 0 | 0 | 0 | 0 | 0 | 0 | 0 | 0 | 0 | 0.01 |
| Streptococcaceae; *Lactococcus* | 0 | 0 | 0.00 | 0.00 | 0 | 0 | 0 | 0 | 0.00 | 0 |
| Streptococcaceae; *Streptococcus* | 0.07 | 0 | 0 | 6.56 | 0 | 0.08 | 0.03 | 0.36 | 0.05 | 0.93 |
| Peptoniphilaceae; *Anaerococcus* | 0 | 0 | 0 | 0 | 0 | 0 | 0 | 0 | 0 | 0.02 |
| Caulobacteraceae; *Phenylobacterium* | 1.15 | 0.00 | 0 | 0 | 0 | 0.01 | 0.01 | 0.01 | 0.00 | 0.01 |
| Bradyrhizobiaceae; *Bradyrhizobium* | 0 | 0 | 0 | 0 | 0 | 0 | 0.00 | 0 | 0.01 | 0 |
| Methylobacteriaceae; *Methylobacterium* | 0 | 0 | 0 | 0 | 0 | 0.01 | 0.01 | 0 | 0 | 0 |
| Acetobacteraceae; *Asaia* | 0.01 | 0 | 0.01 | 0 | 0.01 | 0 | 0 | 0 | 0 | 0 |
| Acetobacteraceae; *Commensalibacter* | 0 | 0 | 0.00 | 3.33 | 8.89 | 0.00 | 0.00 | 0 | 0 | 0 |
| Acetobacteraceae; *Neokomagataea* | 0.00 | 0.00 | 0.00 | 0 | 0.01 | 0.01 | 0 | 0 | 0.00 | 0 |
| Anaplasmataceae; *Wolbachieae* | 0.61 | 0.83 | 0.40 | 0.27 | 0.39 | 0.91 | 0.42 | 0.17 | 0.84 | 0.17 |
| Sphingomonadaceae; *Sphingomonas* | 0.00 | 0 | 0 | 0 | 0.00 | 0.01 | 0 | 0 | 0.00 | 0 |
| Burkholderiaceae; *Burkholderia* | 0.00 | 0 | 0.02 | 0 | 0 | 0 | 0 | 0 | 0 | 0 |
| Desulfovibrionaceae; *Desulfovibrio* | 0 | 0 | 0 | 0.01 | 0 | 0 | 0 | 0 | 0 | 0 |
| Aeromonadaceae; *Aeromonas* | 11.96 | 0.01 | 14.18 | 24.89 | 0.01 | 0.01 | 0.02 | 6.05 | 0.01 | 5.46 |
| Chromatiaceae; *Rhabdochromatium* | 0.00 | 0 | 0 | 0 | 0.02 | 0.03 | 0.10 | 0.02 | 0.03 | 0 |
| Chromatiaceae; *Thiohalocapsa* | 5.75 | 74.11 | 4.16 | 0.25 | 46.28 | 71.87 | 24.58 | 24.72 | 8.08 | 0.01 |
| Granulosicoccaceae; *Granulosicoccus* | 0 | 0 | 0.00 | 0.01 | 1.21 | 0.05 | 0.61 | 0.01 | 0.01 | 0.00 |
| Enterobacteriaceae; *Cedecea* | 0.37 | 0.25 | 0.60 | 0.01 | 0.85 | 0.74 | 0.44 | 0.33 | 1.14 | 0.05 |
| Enterobacteriaceae; *Citrobacter* | 0.07 | 1.30 | 0.00 | 0.00 | 0.09 | 0.04 | 0.04 | 0.01 | 0.00 | 0.01 |
| Enterobacteriaceae; *Cronobacter* | 0 | 0.00 | 0.00 | 0 | 0.01 | 0.13 | 0.00 | 0.20 | 0.76 | 0 |
| Enterobacteriaceae; *Enterobacter* | 0 | 0.00 | 0 | 0 | 0.02 | 0.01 | 0.00 | 0.00 | 0 | 0 |
| Enterobacteriaceae; *Erwinia* | 0.00 | 0 | 0 | 0 | 0.04 | 0 | 0 | 0.05 | 0 | 0 |
| Enterobacteriaceae; *Escherichia* | 4.68 | 2.43 | 0.07 | 0.34 | 17.83 | 18.15 | 21.20 | 5.48 | 87.25 | 0.58 |
| Enterobacteriaceae; *Gibbsiella* | 0.15 | 0.05 | 0.02 | 0.02 | 0.42 | 0.56 | 0.30 | 0.19 | 0.42 | 0 |
| Enterobacteriaceae; *Hafnia* | 0.01 | 0 | 0 | 0 | 0 | 0 | 0 | 0.01 | 0 | 0.00 |
| Enterobacteriaceae; *Klebsiella* | 0.01 | 0.00 | 0.00 | 0.01 | 0.06 | 0.12 | 0.10 | 0.01 | 0.04 | 0 |
| Enterobacteriaceae; *Kluyvera* | 0.16 | 0 | 0.24 | 0.01 | 0 | 0 | 0 | 0.03 | 0 | 0.02 |
| Enterobacteriaceae; *Kosakonia* | 0 | 0 | 0 | 0 | 1.25 | 0.00 | 0 | 0 | 0 | 0.04 |
| Enterobacteriaceae; *Leclercia* | 0 | 0 | 0 | 0 | 0.06 | 0.01 | 0.01 | 0 | 0.01 | 0 |
| Enterobacteriaceae; *Mangrovibacter* | 0 | 0 | 0 | 0.00 | 0 | 0.01 | 0 | 0.07 | 0 | 0 |
| Enterobacteriaceae; *Morganella* | 0.17 | 0.00 | 0 | 0.00 | 0.08 | 0.01 | 0.00 | 0.47 | 0 | 0.00 |
| Enterobacteriaceae; *Pantoea* | 0 | 0 | 0 | 0 | 0.05 | 0.00 | 0.02 | 0 | 0.00 | 0 |
| Enterobacteriaceae; *Pectobacterium* | 0.05 | 0.00 | 0.04 | 0.11 | 17.25 | 0.76 | 9.06 | 0.33 | 0.63 | 0.00 |
| Enterobacteriaceae; *Providencia* | 0.01 | 0 | 0.01 | 0.01 | 0 | 0 | 0 | 0 | 0 | 0 |
| Enterobacteriaceae; *Pseudocitrobacter* | 0 | 0.14 | 0 | 0 | 0.00 | 0.00 | 0.00 | 0 | 0.06 | 0 |
| Enterobacteriaceae; *Raoultella* | 0.02 | 2.87 | 0.02 | 0.01 | 0.04 | 0.03 | 0.02 | 0.01 | 0.00 | 0.00 |
| Enterobacteriaceae; *Rosenbergiella* | 0.03 | 0 | 0.06 | 0.01 | 0 | 0 | 0 | 0.03 | 0 | 0.01 |
| Enterobacteriaceae; *Salmonella* | 0.01 | 0 | 0 | 0.03 | 0 | 0 | 0 | 0.00 | 0 | 0.00 |
| Enterobacteriaceae; *Siccibacter* | 0 | 0.00 | 0.00 | 0 | 0.01 | 0.03 | 0.01 | 0 | 0.01 | 0.00 |
| Enterobacteriaceae; *Trabulsiella* | 0.01 | 0 | 0 | 0 | 0 | 0 | 0 | 0.00 | 0 | 0 |
| Halomonadaceae; *Halomonas* | 1.82 | 0.01 | 5.02 | 0.61 | 0.00 | 0 | 0.01 | 0.01 | 0 | 0.86 |
| Orbaceae; *Gilliamella* | 67.54 | 0.17 | 69.06 | 32.86 | 0.01 | 0.01 | 0.03 | 22.58 | 0.02 | 55.19 |
| Orbaceae; *Orbus* | 0.01 | 0 | 0.00 | 0 | 0 | 0.01 | 0.02 | 0.01 | 0 | 0.01 |
| Pseudomonadaceae; *Pseudomonas* | 0.02 | 0 | 0 | 0 | 0 | 0 | 0 | 0 | 0 | 0 |
| Pseudomonadaceae; *Serpens* | 0 | 0.01 | 0 | 0 | 0 | 0.00 | 0 | 0 | 0.00 | 0 |
| Thiotrichaceae; *Beggiatoa* | 0.01 | 0 | 0 | 0 | 0 | 0.00 | 0 | 0 | 0 | 0 |
| Unassigned | 0.25 | 0.40 | 0.08 | 0.05 | 0.26 | 0.36 | 0.24 | 0.06 | 0.36 | 0.24 |

**Species**

|  | BM3 | BM4 | BM5 | BM6 | BM7 | BU2 | BU3 | BU4 | BU5 | BU6 |
| --- | --- | --- | --- | --- | --- | --- | --- | --- | --- | --- |
| *Corynebacterium*; *Corynebacterium tuberculostearicum* | 0 | 0 | 0 | 0 | 0 | 0.01 | 0.00 | 0 | 0.00 | 0 |
| *Mycobacterium*; *Mycobacterium canariasense* | 0.00 | 0.01 | 0 | 0.00 | 0.01 | 0.00 | 0.01 | 0.01 | 0.00 | 0 |
| *Mycobacterium*; *Mycobacterium intermedium* | 0.07 | 0 | 0 | 0 | 0 | 0 | 0 | 0 | 0 | 0 |
| *Williamsia*; *Williamsia serinedens* | 0.01 | 0 | 0 | 0 | 0 | 0 | 0 | 0 | 0 | 0 |
| *Leifsonia*; *Leifsonia shinshuensis* | 0.01 | 0.01 | 0.01 | 0 | 0.00 | 0.15 | 0.02 | 0.00 | 0.05 | 0.00 |
| *Propionibacterium*; *Propionibacterium acnes*\|*Propionibacterium acnes* KPA171202 | 0.01 | 0 | 0 | 0 | 0 | 0 | 0 | 0 | 0 | 0 |
| *Chthonomonas*; *Chthonomonas calidirosea*\|*Chthonomonas calidirosea* T49 | 0 | 0.00 | 0.01 | 0 | 0 | 0 | 0 | 0 | 0 | 0 |
| *Bacteroides*; *Bacteroides fragilis*\|*Bacteroides fragilis* YCH46 | 0 | 0 | 0.06 | 0.07 | 0.07 | 0.02 | 0.25 | 0.02 | 0.00 | 0 |
| *Dysgonomonas*; *Dysgonomonas gadei*\|*Dysgonomonas gadei* ATCC BAA-286 | 0.00 | 0 | 0 | 0.15 | 0 | 0.32 | 0.03 | 2.75 | 0.08 | 0.16 |
| *Dysgonomonas*; *Dysgonomonas macrotermitis* | 0 | 17.24 | 0 | 0 | 0 | 0 | 0 | 0 | 0 | 0 |
| Microbacter; Microbacter margulisiae | 0 | 0.12 | 0 | 0 | 0 | 0 | 0 | 0 | 0 | 0 |
| *Parabacteroides*; *Parabacteroides chartae* | 3.72 | 0 | 0 | 0 | 0 | 0 | 0 | 0 | 0.00 | 0 |
| *Porphyromonas*; *Porphyromonas cangingivalis* | 0 | 0 | 0.00 | 0.02 | 0 | 0 | 0 | 0.00 | 0 | 6.67 |
| *Porphyromonas*; *Porphyromonas catoniae*\|*Porphyromonas catoniae* ATCC 51270 | 0 | 0 | 0.01 | 0.00 | 0 | 0 | 0 | 0 | 0 | 0 |
| *Porphyromonas*; *Porphyromonas crevioricanis* | 0 | 0 | 0.05 | 0.01 | 0.01 | 0.01 | 0.06 | 0.01 | 0.00 | 0 |
| *Chishuiella*; *Chishuiella changwenlii* | 0 | 0 | 0.02 | 0 | 0.01 | 0 | 0.01 | 0 | 0 | 0 |
| *Chryseobacterium*; *Chryseobacterium aquifrigidense* | 0.00 | 0.01 | 4.25 | 5.26 | 4.69 | 1.55 | 26.68 | 1.01 | 0.07 | 0.00 |
| *Chryseobacterium*; *Chryseobacterium vietnamense* | 0 | 0 | 0.02 | 0.00 | 0 | 0 | 0 | 0 | 0 | 0 |
| *Flavobacterium*; *Flavobacterium fontis* | 0 | 0 | 0.07 | 0.01 | 0.02 | 0.01 | 0.10 | 0 | 0.00 | 0 |
| *Flavobacterium*; *Flavobacterium indicum*\|*Flavobacterium indicum* GPTSA100-9 = DSM 17447 | 0 | 0 | 0.02 | 0 | 0 | 0 | 0 | 0 | 0 | 0 |
| *Flavobacterium*; *Flavobacterium squillarum* | 0 | 0 | 0.00 | 0.01 | 0.01 | 0.01 | 0.03 | 0.00 | 0 | 0 |
| *Mastigocoleus*; *Mastigocoleus testarum* | 0.03 | 0.01 | 0.13 | 0 | 0.00 | 0.00 | 0.01 | 0.00 | 0.00 | 0.00 |
| *Enterococcus*; *Enterococcus moraviensis* | 0 | 0 | 0.00 | 0.00 | 0 | 0 | 0 | 0 | 0.00 | 0 |
| *Enterococcus*; *Enterococcus raffinosus* | 1.17 | 0.01 | 1.31 | 24.99 | 0.01 | 3.45 | 15.40 | 34.86 | 0.06 | 29.45 |
| *Enterococcus*; *Enterococcus termitis* | 0.01 | 0 | 0.01 | 0 | 0 | 0.21 | 0 | 0.01 | 0 | 0 |
| *Vagococcus*; *Vagococcus penaei* | 0.00 | 0 | 0.00 | 0.00 | 0 | 0.27 | 0.06 | 0.06 | 0 | 0.00 |
| *Leuconostoc*; *Leuconostoc pseudomesenteroides*\|*Leuconostoc pseudomesenteroides* KCTC 3652 | 0.07 | 0 | 0 | 6.55 | 0 | 0.07 | 0.02 | 0.36 | 0.03 | 0.93 |
| *Lactococcus*; *Lactococcus lactis*\|*Lactococcus lactis subsp. lactis*\|*Lactococcus lactis subsp. lactis* Il1403 | 0 | 0 | 0 | 0.00 | 0 | 0.01 | 0.01 | 0 | 0.02 | 0 |
| *Lactococcus*; *Lactococcus plantarum* | 0 | 0 | 0 | 0 | 0 | 0 | 0 | 0 | 0 | 0.01 |
| *Streptococcus*; *Streptococcus dentirousetti* | 0 | 0 | 0 | 0 | 0 | 0 | 0 | 0 | 0 | 0.02 |
| *Anaerococcus*; *Anaerococcus senegalensis*\|*Anaerococcus senegalensis* JC48 | 0.01 | 0.00 | 0.02 | 0.08 | 0 | 0.01 | 0.03 | 0.03 | 0 | 0.06 |
| *Phenylobacterium*; *Phenylobacterium koreense* | 0 | 0 | 0 | 0 | 0.01 | 0 | 0 | 0 | 0 | 0 |
| *Bradyrhizobium*; *Bradyrhizobium ottawaense* | 0.01 | 0 | 0.01 | 0 | 0 | 0 | 0 | 0 | 0 | 0 |
| *Methylobacterium*; *Methylobacterium phyllostachyos* | 1.15 | 0.00 | 0 | 0 | 0 | 0.01 | 0.01 | 0.01 | 0.00 | 0.01 |
| *Asaia*; *Asaia krungthepensis* | 0.01 | 0 | 0 | 0 | 0 | 0.00 | 0 | 0 | 0 | 0 |
| *Commensalibacter*; *Commensalibacter intestini*\|*Commensalibacter intestini* A911 | 0.00 | 0.00 | 0.00 | 0 | 0.01 | 0.01 | 0 | 0 | 0.00 | 0 |
| *Neokomagataea*; *Neokomagataea tanensis*\|*Neokomagataea tanensis* NBRC 106556 | 0.61 | 0.83 | 0.40 | 0.27 | 0.39 | 0.91 | 0.42 | 0.17 | 0.84 | 0.17 |
| *Wolbachieae*; *Wolbachia*\|*Wolbachia* endosymbiont of *Culex quinquefasciatus*\|*Wolbachia* endosymbiont of *Culex quinquefasciatus* Pel | 0.00 | 0 | 0.00 | 0 | 0 | 0 | 0 | 0.00 | 0 | 0.00 |
| *Wolbachieae*; *Wolbachia*\|*Wolbachia* sp. wRi | 0.00 | 0 | 0 | 0 | 0.02 | 0.03 | 0.10 | 0.02 | 0.03 | 0 |
| *Sphingomonas*; *Sphingomonas* *echinoides* | 0 | 0.01 | 0 | 0 | 0.01 | 0.04 | 0.00 | 0.08 | 0 | 0 |
| *Burkholderia*; *Burkholderia* *cepacia* complex\|*Burkholderia lata* | 5.72 | 73.43 | 4.12 | 0.24 | 46.13 | 68.79 | 24.49 | 24.61 | 7.75 | 0.01 |
| *Burkholderia*; *pseudomallei* group\|*Burkholderia thailandensis* | 0 | 0.07 | 0 | 0 | 0.01 | 0.00 | 0.00 | 0.00 | 0 | 0 |
| *Desulfovibrio*; *Desulfovibrio arcticus* | 0.03 | 0.58 | 0 | 0.00 | 0.11 | 0.11 | 0.06 | 0.02 | 0.15 | 0 |
| *Desulfovibrio*; *Desulfovibrio cuneatus* | 0 | 0.00 | 0 | 0 | 0.01 | 0 | 0.01 | 0 | 0.00 | 0 |
| *Desulfovibrio*; *Desulfovibrio desulfuricans*\|*Desulfovibrio desulfuricans* subsp. *desulfuricans*\|*Desulfovibrio* *desulfuricans* subsp. *desulfuricans* str. ATCC 27774 | 0.00 | 0.01 | 0.03 | 0 | 0.00 | 0.02 | 0.01 | 0.01 | 0.01 | 0 |
| *Desulfovibrio*; *Desulfovibrio inopinatus* | 0 | 0 | 0 | 0 | 0.00 | 0.02 | 0 | 0 | 0 | 0 |
| *Desulfovibrio*; *Desulfovibrio intestinalis* | 0 | 0 | 0 | 0 | 0.01 | 2.88 | 0.00 | 0.01 | 0.16 | 0 |
| *Desulfovibrio*; *Desulfovibrio legallii* | 0 | 0 | 0 | 0 | 0 | 0 | 0.00 | 0 | 0.01 | 0 |
| *Desulfovibrio*; *Desulfovibrio litoralis* | 0 | 0 | 0 | 0 | 0.02 | 0.00 | 0.03 | 0 | 0.00 | 0 |
| *Desulfovibrio*; *Desulfovibrio oxamicus* | 0 | 0 | 0.00 | 0.01 | 0.91 | 0.01 | 0.47 | 0.01 | 0 | 0.00 |
| *Desulfovibrio*; *Desulfovibrio simplex* | 0 | 0 | 0 | 0 | 0 | 0.02 | 0 | 0 | 0 | 0 |
| *Desulfovibrio*; *Desulfovibrio vulgaris*\|*Desulfovibrio vulgaris* DP4 | 0 | 0 | 0 | 0 | 0.26 | 0.02 | 0.10 | 0.00 | 0.01 | 0 |
| *Aeromonas*; *Aeromonas hydrophila* | 0 | 0 | 0 | 0 | 0.01 | 0 | 0.01 | 0 | 0 | 0 |
| *Aeromonas*; *Aeromonas sobria* | 0 | 0 | 0.01 | 0 | 0 | 0 | 0 | 0.01 | 0 | 0 |
| *Rhabdochromatium*; *Rhabdochromatium marinum* | 8.55 | 0.01 | 13.50 | 23.40 | 0.00 | 0.00 | 0.01 | 4.77 | 0.00 | 4.10 |
| *Thiohalocapsa*; *Thiohalocapsa marina* | 0.04 | 0 | 0.11 | 0.01 | 0 | 0 | 0 | 0.01 | 0 | 0.02 |
| *Granulosicoccus*; *Granulosicoccus antarcticus*\|*Granulosicoccus antarcticus* IMCC3135 | 0.01 | 0 | 0.00 | 0 | 0 | 0 | 0 | 0.04 | 0 | 0.01 |
| *Granulosicoccus*; *Granulosicoccus marinus* | 0.01 | 0 | 0 | 0.02 | 0 | 0 | 0 | 0.00 | 0 | 0.00 |
| *Cedecea*; *Cedecea lapagei* | 0 | 0 | 0 | 0.04 | 0 | 0 | 0 | 0.01 | 0 | 0.00 |
| *Citrobacter*; *Citrobacter freundii complex*\|*Citrobacter braakii* | 0.30 | 0 | 0.19 | 0.22 | 0 | 0 | 0 | 0.96 | 0 | 0.53 |
| *Citrobacter*; *Citrobacter freundii complex*\|*Citrobacter freundii* | 2.47 | 0 | 0.35 | 0.44 | 0.00 | 0 | 0.01 | 0.07 | 0 | 0.65 |
| *Citrobacter*; *Citrobacter freundii complex*\|*Citrobacter gillenii* | 0.26 | 0 | 0.02 | 0.42 | 0 | 0.00 | 0 | 0.15 | 0.00 | 0.11 |
| *Citrobacter*; *Citrobacter freundii complex*\|*Citrobacter murliniae* | 0.33 | 0 | 0 | 0.34 | 0 | 0 | 0 | 0.04 | 0 | 0.04 |
| *Citrobacter*; *Citrobacter freundii complex*\|*Citrobacter sedlakii* | 0.02 | 0.02 | 0.02 | 0.00 | 0.26 | 0.17 | 0.06 | 0.03 | 0.06 | 0.00 |
| *Citrobacter*; *Citrobacter freundii complex*\|*Citrobacter werkmanii* | 0.07 | 0.00 | 0.03 | 0 | 0.26 | 0.17 | 0.13 | 0.07 | 0.08 | 0.00 |
| *Citrobacter*; *Citrobacter koseri* | 0.27 | 0.12 | 0.55 | 0.01 | 0.29 | 0.36 | 0.22 | 0.23 | 0.97 | 0.05 |
| *Citrobacter*; *Citrobacter koseri*\|*Citrobacter koseri* ATCC BAA-895 | 0.00 | 0.03 | 0 | 0 | 0.02 | 0.00 | 0.01 | 0.00 | 0.03 | 0 |
| *Cronobacter*; *Cronobacter dublinensis* | 0.00 | 0.07 | 0 | 0 | 0.02 | 0.03 | 0.02 | 0.01 | 0.00 | 0.00 |
| *Cronobacter*; *Cronobacter dublinensis*\|*Cronobacter dublinensis* subsp. *lactaridi* | 0.02 | 0 | 0 | 0 | 0 | 0 | 0 | 0 | 0 | 0.00 |
| *Cronobacter*; *Cronobacter muytjensii*\|*Cronobacter muytjensii* ATCC 51329 | 0.05 | 0 | 0 | 0.00 | 0.04 | 0.00 | 0.00 | 0 | 0 | 0.00 |
| *Cronobacter*; *Cronobacter sakazakii*\|*Cronobacter sakazakii* ATCC BAA-894 | 0 | 0 | 0 | 0 | 0.03 | 0 | 0.01 | 0 | 0 | 0 |
| *Cronobacter*; *Cronobacter turicensis*\|*Cronobacter turicensis* z3032 | 0.00 | 1.30 | 0.00 | 0 | 0.02 | 0.04 | 0.02 | 0.01 | 0.00 | 0.00 |
| *Enterobacter*; *Enterobacter aerogenes*\|*Enterobacter aerogenes* KCTC 2190 | 0 | 0.00 | 0.00 | 0 | 0.01 | 0.13 | 0.00 | 0.20 | 0.76 | 0 |
| *Enterobacter*; *Enterobacter cloacae* complex\|*Enterobacter* *asburiae*\|*Enterobacter asburiae* LF7a | 0 | 0.00 | 0 | 0 | 0.02 | 0.01 | 0.00 | 0.00 | 0 | 0 |
| *Enterobacter*; *Enterobacter cloacae* complex\|*Enterobacter cloacae* | 1.82 | 0.01 | 5.02 | 0.61 | 0.00 | 0 | 0.01 | 0.01 | 0 | 0.86 |
| *Enterobacter*; *Enterobacter cloacae* complex\|*Enterobacter cloacae*\|*Enterobacter cloacae* subsp. *dissolvens* | 0.01 | 0 | 0 | 0.02 | 0 | 0 | 0 | 0 | 0 | 0 |
| *Enterobacter*; *Enterobacter* sp. 638 | 0 | 0 | 0 | 0.01 | 0 | 0 | 0 | 0.00 | 0 | 0.00 |
| *Erwinia*; *Erwinia aphidicola* | 0 | 0 | 0 | 0 | 0 | 0 | 0 | 0.03 | 0 | 0 |
| *Erwinia*; *Erwinia persicina* | 0.00 | 0 | 0 | 0 | 0.04 | 0 | 0 | 0.02 | 0 | 0 |
| *Erwinia*; *Erwinia psidii* | 0 | 0.00 | 0.00 | 0 | 0.01 | 0.03 | 0.01 | 0 | 0.01 | 0.00 |
| *Erwinia*; *Erwinia pyrifoliae*\|*Erwinia pyrifoliae* Ep1/96 | 4.62 | 2.18 | 0.04 | 0.33 | 16.01 | 16.76 | 19.98 | 5.25 | 86.25 | 0.52 |
| *Escherichia*; *Escherichia fergusonii*\|*Escherichia fergusonii* ATCC 35469 | 0 | 0.01 | 0.00 | 0 | 0.05 | 0.05 | 0.03 | 0.03 | 0.20 | 0.00 |
| *Gibbsiella*; *Gibbsiella greigii* | 0.00 | 0.00 | 0.01 | 0.00 | 0.49 | 0.45 | 0.39 | 0.07 | 0.16 | 0.05 |
| *Hafnia*; *Hafnia alvei* | 0.00 | 0 | 0.00 | 0.00 | 0.06 | 0.09 | 0.06 | 0.05 | 0.07 | 0.01 |
| *Hafnia*; *Hafnia paralvei* | 0.03 | 0.23 | 0.02 | 0.00 | 0.07 | 0.08 | 0.05 | 0.02 | 0.14 | 0 |
| *Klebsiella*; *Klebsiella oxytoca* | 0.02 | 0.01 | 0 | 0.00 | 1.14 | 0.69 | 0.68 | 0.06 | 0.43 | 0.01 |
| *Klebsiella*; *Klebsiella oxytoca*\|*Klebsiella oxytoca* KCTC 1686 | 0 | 0.00 | 0 | 0.00 | 0.01 | 0.02 | 0.01 | 0.00 | 0.01 | 0 |
| *Klebsiella*; *Klebsiella pneumoniae*\|*Klebsiella pneumoniae* subsp. *pneumoniae*\|*Klebsiella pneumoniae* subsp. *pneumoniae* MGH 78578 | 0.15 | 0.05 | 0.02 | 0.02 | 0.40 | 0.56 | 0.26 | 0.19 | 0.42 | 0 |
| *Klebsiella*; *Klebsiella quasipneumoniae*\|*Klebsiella quasipneumoniae* subsp. *quasipneumoniae* | 0 | 0 | 0 | 0 | 0.02 | 0.01 | 0.05 | 0 | 0.00 | 0 |
| *Klebsiella*; *Klebsiella quasipneumoniae*\|*Klebsiella quasipneumoniae* subsp. *similipneumoniae* | 0.01 | 0 | 0 | 0 | 0 | 0 | 0 | 0.01 | 0 | 0.00 |
| *Klebsiella*; *Klebsiella variicola* | 0.01 | 0.00 | 0.00 | 0.01 | 0.06 | 0.12 | 0.10 | 0.01 | 0.04 | 0 |
| *Klebsiella*; *Klebsiella variicola*\|*Klebsiella variicola* At-22 | 0.16 | 0 | 0.24 | 0.01 | 0 | 0 | 0 | 0.03 | 0 | 0.02 |
| *Kluyvera*; *Kluyvera cryocrescens* | 0.01 | 0 | 0 | 0 | 0 | 0 | 0 | 0.00 | 0 | 0 |
| *Kluyvera*; *Kluyvera intermedia* | 0 | 0 | 0 | 0 | 1.25 | 0.00 | 0 | 0 | 0 | 0.04 |
| *Kosakonia*; *Kosakonia sacchari* | 0 | 0 | 0 | 0 | 0 | 0.01 | 0.01 | 0 | 0 | 0 |
| *Leclercia*; *Leclercia adecarboxylata* | 0.00 | 0 | 0.01 | 32.04 | 0.00 | 0.00 | 0.02 | 22.44 | 0.00 | 0.29 |
| *Mangrovibacter*; *Mangrovibacter plantisponsor* | 67.54 | 0.17 | 69.06 | 0.81 | 0.01 | 0.01 | 0.01 | 0.14 | 0.01 | 54.90 |
| *Morganella*; *Morganella* *morganii*\|*Morganella morganii* subsp. *morganii*\|*Morganella morganii* subsp. *morganii* KT | 0 | 0 | 0 | 0 | 0.00 | 0.01 | 0.01 | 0 | 0.01 | 0 |
| *Pantoea*; *Pantoea dispersa* | 0 | 0 | 0 | 0 | 0.01 | 0 | 0.00 | 0 | 0 | 0 |
| *Pantoea*; *Pantoea eucalypti* | 0 | 0 | 0 | 0 | 0.01 | 0 | 0.00 | 0 | 0 | 0 |
| *Pantoea*; *Pantoea stewartii*\|*Pantoea stewartii* subsp. *indologenes* | 0 | 0 | 0 | 0 | 0.03 | 0 | 0.00 | 0 | 0 | 0 |
| *Pantoea*; *Pantoea wallisii* | 0 | 0 | 0 | 0.00 | 0 | 0 | 0 | 0.07 | 0 | 0 |
| *Pectobacterium*; *Pectobacterium atrosepticum*\|*Pectobacterium atrosepticum* SCRI1043 | 0 | 0 | 0 | 0 | 0 | 0.01 | 0 | 0 | 0 | 0 |
| *Pectobacterium*; *Pectobacterium carotovorum*\|*Pectobacterium carotovorum* subsp. *actinidiae* | 0.00 | 0 | 0 | 0 | 0.00 | 0.01 | 0 | 0 | 0.00 | 0 |
| *Providencia*; *Providencia alcalifaciens*\|*Providencia alcalifaciens* DSM 30120 | 0.14 | 0.00 | 0 | 0.00 | 0.08 | 0.01 | 0.00 | 0.47 | 0 | 0.00 |
| *Providencia*; *Providencia* *stuartii*\|*Providencia stuartii* MRSN 2154 | 0.04 | 0 | 0 | 0 | 0 | 0 | 0 | 0 | 0 | 0 |
| *Pseudocitrobacter*; *Pseudocitrobacter anthropi* | 0 | 0 | 0 | 0 | 0.05 | 0.00 | 0.02 | 0 | 0.00 | 0 |
| *Raoultella*; *Raoultella electrica* | 0 | 0 | 0.00 | 0 | 0 | 0.01 | 0.00 | 0 | 0 | 0 |
| *Raoultella*; *Raoultella ornithinolytica* | 0 | 0 | 0 | 0 | 0 | 0 | 0 | 0.01 | 0 | 0.01 |
| *Raoultella*; *Raoultella ornithinolytica*\|*Raoultella ornithinolytica* B6 | 0.01 | 0 | 0 | 0 | 0 | 0 | 0.00 | 0.00 | 0 | 0 |
| *Raoultella*; *Raoultella planticola*\|*Raoultella planticola* ATCC 33531 | 0 | 0 | 0 | 0 | 0 | 0 | 0.02 | 0 | 0 | 0 |
| *Raoultella*; *Raoultella terrigena* | 0 | 0 | 0 | 0 | 0.04 | 0 | 0.03 | 0 | 0 | 0 |
| *Rosenbergiella*; *Rosenbergiella australoborealis* | 0 | 0 | 0.00 | 0 | 0 | 0 | 0 | 0.02 | 0 | 0 |
| *Salmonella*; *Salmonella enterica*\|*Salmonella enterica* subsp. *arizonae* | 0 | 0 | 0 | 0 | 0 | 0.02 | 0 | 0 | 0 | 0 |
| *Salmonella*; *Salmonella enterica*\|*Salmonella enterica* subsp*. enterica*\|*Salmonella enterica* subsp*. enterica serovar* Typhimurium | 0.00 | 0.00 | 0.00 | 0.11 | 17.15 | 0.68 | 9.01 | 0.29 | 0.62 | 0.00 |
| *Salmonella*; *Salmonella subterranea* | 0.05 | 0 | 0.03 | 0 | 0.06 | 0.07 | 0.02 | 0.03 | 0.01 | 0 |
| *Siccibacter*; *Siccibacter colletis* | 0.00 | 0 | 0.02 | 0 | 0 | 0 | 0 | 0 | 0 | 0 |
| *Siccibacter*; *Siccibacter turicensis* | 0.01 | 0 | 0.01 | 0.01 | 0 | 0 | 0 | 0 | 0 | 0 |
| *Siccibacter*; *Siccibacter turicensis*\|*Siccibacter turicensis* LMG 23730 | 0 | 0 | 0 | 0 | 0 | 0 | 0 | 0 | 0.02 | 0 |
| *Trabulsiella*; *Trabulsiella guamensis* | 0 | 0.00 | 0 | 0 | 0.00 | 0.00 | 0.00 | 0 | 0.04 | 0 |
| *Halomonas*; *Halomonas stevensii*\|*Halomonas stevensii* S18214 | 0 | 0.14 | 0 | 0 | 0 | 0 | 0 | 0 | 0 | 0 |
| *Gilliamella*; *Gilliamella apicola* | 0.02 | 0 | 0 | 0 | 0 | 0 | 0 | 0 | 0 | 0 |
| *Orbus*; *Orbus hercynius* | 0.01 | 0 | 0.01 | 0 | 0 | 0 | 0 | 0 | 0 | 0 |
| *Orbus*; *Orbus sasakiae* | 0 | 0.01 | 0.00 | 0.01 | 0 | 0.00 | 0 | 0.01 | 0 | 0 |
| *Pseudomonas*; *Pseudomonas aeruginosa* group\|*Pseudomonas aeruginosa* | 0.01 | 2.85 | 0.01 | 0 | 0.04 | 0.03 | 0.02 | 0.00 | 0.00 | 0.00 |
| *Pseudomonas*; *Pseudomonas composti* | 0 | 0.01 | 0 | 0 | 0 | 0.00 | 0 | 0 | 0.00 | 0 |
| *Pseudomonas*; *Pseudomonas graminis* | 0 | 0 | 0 | 0.01 | 0 | 0 | 0 | 0 | 0 | 0 |
| *Pseudomonas*; *Pseudomonas knackmussii*\|*Pseudomonas knackmussii* B13 | 0.03 | 0 | 0.06 | 0.01 | 0 | 0 | 0 | 0.03 | 0 | 0.01 |
| *Serpens*; *Serpens flexibilis* | 0 | 0 | 0.00 | 3.31 | 8.87 | 0.00 | 0.00 | 0 | 0 | 0 |
| *Beggiatoa*; *Beggiatoa alba*\|*Beggiatoa alba* B18LD | 0 | 0 | 0 | 0.02 | 0.02 | 0 | 0 | 0 | 0 | 0 |
| Unassigned | 0.25 | 0.40 | 0.08 | 0.05 | 0.26 | 0.36 | 0.24 | 0.06 | 0.36 | 0.24 |

**Supplementary Table S2** Number of bacterial OTUs detected in the samples of *Bactrocera melastomatos* and *Bactrocera umbrosa* from Peninsular Malaysia

| OTU | Total | *B. melastomatos*  (Awana) | *B. melastomtos*  (U. Malaya) | *B. umbrosa*  (U. Malaya) |
| --- | --- | --- | --- | --- |
| Phylum | 6 | 6 | 5 | 5 |
| Class | 11 | 11 | 10 | 10 |
| Order | 23 | 23 | 17 | 21 |
| Family | 30 | 28 | 20 | 24 |
| Genus | 64 | 54 | 47 | 54 |
| Species | 122 | 94 | 85 | 102 |

**Supplementary Table S3** Relative abundance (mean ± SD) of the bacterial OTUs in field-caught adult male flies of *Bactrocera melastomatos* and *Bactrocera umbrosa* determined by 16S rRNA gene sequencing and one-way ANOVA test on differences between the means of the samples from Peninsular Malaysia. ^*^significant difference; ^#^not significant by Tukey HSD Test.

| Taxonomy | BM Awana | BM Universiti Malaya | BU Universiti Malaya | ANOVA | |
| --- | --- | --- | --- | --- | --- |
| **p: Actinobacteria** | 0.04±0.05  (0.01 - 0.1) | 0.01±0.01  (0.00 - 0.01) | 0.05±0.06 (0.00 - 0.16) | F=0.438 | p=0.662 |
| c: Actinobacteria | 0.04±0.05  (0.01 - 0.1) | 0.01±0.01  (0.00 - 0.01) | 0.05±0.06 (0.00 - 0.16) | F=0.438 | p=0.662 |
| o: Corynebacteriales | 0.03±0.05  (0 - 0.09) | 0  (0) | 0.00±0.00  (0 - 0.01) | F=1.074 | p=0.392 |
| f: Corynebacteriaceae | 0  (0) | 0  (0) | 0.00±0.00  (0 - 0.01) | F=1.133 | p=0.375 |
| g: *Corynebacterium* | 0  (0) | 0  (0) | 0.00±0.00  (0 - 0.01) | F=1.133 | p=0.375 |
| s: *Corynebacterium tuberculostearicum* | 0  (0) | 0  (0) | 0.00±0.00  (0 - 0.01) | F=1.133 | p=0.375 |
| f: Mycobacteriaceae | 0.03±0.05  (0 - 0.08) | 0  (0) | 0  (0) | F=1.225 | p=0.35 |
| g: *Mycobacterium* | 0.03±0.05  (0 - 0.08) | 0  (0) | 0  (0) | F=1.225 | p=0.35 |
| s: *Mycobacterium canariasense* | 0.02±0.04  (0 - 0.07) | 0  (0) | 0  (0) | F=1.225 | p=0.35 |
| s: *Mycobacterium intermedium* | 0.00±0.01  (0 - 0.01) | 0  (0) | 0  (0) | F=1.225 | p=0.35 |
| f: Williamsiaceae | 0.00±0.01  (0 - 0.01) | 0  (0) | 0  (0) | F=1.225 | p=0.35 |
| g: *Williamsia* | 0.00±0.01  (0 - 0.01) | 0  (0) | 0  (0) | F=1.225 | p=0.35 |
| s: *Williamsia serinedens* | 0.00±0.01  (0 - 0.01) | 0  (0) | 0  (0) | F=1.225 | p=0.35 |
| o: Micrococcales | 0.00±0.00  (0 - 0.01) | 0.01±0.01  (0.00 - 0.01) | 0.00±0.00  (0 - 0.01) | F=0.422 | p=0.671 |
| f: Microbacteriaceae | 0.00±0.00  (0 - 0.01) | 0.01±0.01  (0.00 - 0.01) | 0.00±0.00  (0 - 0.01) | F=0.422 | p=0.671 |
| g: *Leifsonia* | 0.00±0.00  (0 - 0.01) | 0.01±0.01  (0.00 - 0.01) | 0.00±0.00  (0 - 0.01) | F=0.422 | p=0.671 |
| s: *Leifsonia shinshuensis* | 0.00±0.00  (0 - 0.01) | 0.01±0.01  (0.00 - 0.01) | 0.00±0.00  (0 - 0.01) | F=0.422 | p=0.671 |
| o: Propionibacteriales | 0.01±0.00  (0.01 - 0.01) | 0.00±0.00 (0 - 0.00) | 0.04±0.06 (0.00 - 0.15) | F=0.996 | p=0.416 |
| f: Propionibacteriaceae | 0.01±0.00  (0.01 - 0.01) | 0.00±0.00 (0 - 0.00) | 0.04±0.06 (0.00 - 0.15) | F=0.996 | p=0.416 |
| g: *Propionibacterium* | 0.01±0.00  (0.01 - 0.01) | 0.00±0.00 (0 - 0.00) | 0.04±0.06 (0.00 - 0.15) | F=0.996 | p=0.416 |
| s: *Propionibacterium acnes*  \|*Propionibacterium acnes* KPA171202 | 0.01±0.00  (0.01 - 0.01) | 0.00±0.00 (0 - 0.00) | 0.04±0.06 (0.00 - 0.15) | F=0.996 | p=0.416 |
| **p: Armatimonadetes** | 0.00±0.00  (0 - 0.01) | 0 (0) | 0 (0) | F=1.959 | p=0.211 |
| c: Chthonomonadetes | 0.00±0.00  (0 - 0.01) | 0 (0) | 0 (0) | F=1.959 | p=0.211 |
| o: Chthonomonadales | 0.00±0.00  (0 - 0.01) | 0 (0) | 0 (0) | F=1.959 | p=0.211 |
| f: Chthonomonadaceae | 0.00±0.00  (0 - 0.01) | 0 (0) | 0 (0) | F=1.959 | p=0.211 |
| g: *Chthonomonas* | 0.00±0.00  (0 - 0.01) | 0 (0) | 0 (0) | F=1.959 | p=0.211 |
| s: *Chthonomonas calidirosea*  \|*Chthonomonas calidirosea* T49 | 0.00±0.00  (0 - 0.01) | 0 (0) | 0 (0) | F=1.959 | p=0.211 |
| **p: Bacteroidetes** | 8.54±7.66  (3.72 - 17.37) | 5.18±0.51 (4.82 - 5.54) | 7.98±11.01 (0.16 - 27.17) | F=0.088 | p=0.917 |
| c: Bacteroidia | 2.72±2.38  (0.01 - 4.44) | 5.09±0.41 (4.8 - 5.38) | 7.29±11.35 (0.07 - 27.07) | F=0.263 | p=0.776 |
| o: Bacteroidales | 2.72±2.38  (0.01 - 4.44) | 5.09±0.41 (4.8 - 5.38) | 7.29±11.35 (0.07 - 27.07) | F=0.263 | p=0.776 |
| f: Bacteroidaceae | 0.02±0.04  (0 - 0.06) | 0.07±0.00 (0.07 - 0.07) | 0.06±0.11 (0 - 0.25) | F=0.283 | p=0.762 |
| g: *Bacteroides* | 0.02±0.04  (0 - 0.06) | 0.07±0.00 (0.07 - 0.07) | 0.06±0.11 (0 - 0.25) | F=0.283 | p=0.762 |
| s: *Bacteroides fragilis*\|*Bacteroides fragilis* YCH46 | 0.02±0.04  (0 - 0.06) | 0.07±0.00 (0.07 - 0.07) | 0.06±0.11 (0 - 0.25) | F=0.283 | p=0.762 |
| f: Porphyromonadaceae | 2.7±2.36  (0.01 - 4.38) | 5.02±0.41 (4.73 - 5.31) | 7.23±11.24 (0.07 - 26.81) | F=0.263 | p=0.776 |
| g: *Dysgonomonas* | 1.24±2.15  (0 - 3.72) | 0.01±0.02 (0 - 0.02) | 1.33±2.98 (0 - 6.67) | F=0.209 | p=0.817 |
| s: *Dysgonomonas gadei*\|*Dysgonomonas gadei* ATCC BAA-286 | 1.24±2.15  (0 - 3.72) | 0 (0) | 0.00±0.00 (0 - 0.00) | F=1.225 | p=0.35 |
| s: *Dysgonomonas macrotermitis* | 0.00±0.00  (0 - 0.00) | 0.01±0.02 (0 - 0.02) | 1.33±2.98 (0 - 6.67) | F=0.435 | p=0.664 |
| g: *Microbacter* | 1.42±2.45  (0.00 - 4.25) | 4.98±0.4 (4.69 - 5.26) | 5.86±11.66 (0.00 - 26.68) | F=0.239 | p=0.794 |
| s: *Microbacter margulisiae* | 1.42±2.45  (0.00 - 4.25) | 4.98±0.4 (4.69 - 5.26) | 5.86±11.66 (0.00 - 26.68) | F=0.239 | p=0.794 |
| g: *Parabacteroides* | 0.01±0.01  (0 - 0.02) | 0.00±0.00 (0 - 0.00) | 0 (0) | F=1.103 | p=0.384 |
| s: *Parabacteroides chartae* | 0.01±0.01  (0 - 0.02) | 0.00±0.00 (0 - 0.00) | 0 (0) | F=1.103 | p=0.384 |
| g: *Porphyromonas* | 0.03±0.06  (0 - 0.1) | 0.03±0.01 (0.02 - 0.03) | 0.03±0.06 (0 - 0.13) | F=0.007 | p=0.993 |
| s: *Porphyromonas cangingivalis* | 0.02±0.04  (0 - 0.07) | 0.02±0.01 (0.01 - 0.02) | 0.02±0.04 (0 - 0.1) | F=0.011 | p=0.989 |
| s: *Porphyromonas catoniae*  \|*Porphyromonas catoniae* ATCC 51270 | 0.01±0.01  (0 - 0.02) | 0 (0) | 0 (0) | F=1.225 | p=0.35 |
| s: *Porphyromonas crevioricanis* | 0.00±0.00  (0 - 0.00) | 0.01±0.00 (0.01 - 0.01) | 0.01±0.01 (0 - 0.03) | F=0.486 | p=0.635 |
| c: Flavobacteriia | 5.81±10  (0.00 - 17.36) | 0.09±0.1 (0.02 - 0.16) | 0.69±1.16 (0.09 - 2.76) | F=1.013 | p=0.411 |
| o: Flavobacteriales | 5.81±10  (0.00 - 17.36) | 0.09±0.1 (0.02 - 0.16) | 0.69±1.16 (0.09 - 2.76) | F=1.013 | p=0.411 |
| f: Flavobacteriaceae | 5.81±10  (0.00 - 17.36) | 0.09±0.1 (0.02 - 0.16) | 0.69±1.16 (0.09 - 2.76) | F=1.013 | p=0.411 |
| g: *Chishuiella* | 0.00±0.00  (0 - 0.00) | 0.07±0.1 (0 - 0.15) | 0.67±1.17 (0.03 - 2.75) | F=0.659 | p=0.547 |
| s: *Chishuiella changwenlii* | 0.00±0.00  (0 - 0.00) | 0.07±0.1 (0 - 0.15) | 0.67±1.17 (0.03 - 2.75) | F=0.659 | p=0.547 |
| g: *Chryseobacterium* | 5.79±10.02  (0 - 17.36) | 0 (0) | 0 (0) | F=1.225 | p=0.35 |
| s: *Chryseobacterium* *aquifrigidense* | 5.75±9.96  (0 - 17.24) | 0 (0) | 0 (0) | F=1.225 | p=0.35 |
| s: *Chryseobacterium vietnamense* | 0.04±0.07  (0 - 0.12) | 0 (0) | 0 (0) | F=1.225 | p=0.35 |
| g: *Flavobacterium* | 0.03±0.05  (0 - 0.08) | 0.02±0.00 (0.02 - 0.02) | 0.02±0.03 (0 - 0.07) | F=0.082 | p=0.922 |
| s: *Flavobacterium fontis* | 0.00±0.00  (0 - 0.01) | 0.00±0.00 (0 - 0.00) | 0 (0) | F=1.062 | p=0.396 |
| s: *Flavobacterium indicum*  \|*Flavobacterium indicum* GPTSA100-9 = DSM 17447 | 0.02±0.03  (0 - 0.05) | 0.01±0.00 (0.01 - 0.01) | 0.02±0.03 (0 - 0.06) | F=0.017 | p=0.983 |
| s: *Flavobacterium squillarum* | 0.01±0.01 (0 - 0.02) | 0.00±0.00 (0 - 0.01) | 0.00±0.00 (0 - 0.01) | F=0.453 | p=0.653 |
| **p: Cyanobacteria/Melainabacteria group** | 0.05±0.06 (0.01 - 0.13) | 0.00±0.00 (0 - 0.00) | 0.00±0.00 (0.00 - 0.01) | F=0.94 | p=0.435 |
| c: Cyanobacteria | 0.05±0.06 (0.01 - 0.13) | 0.00±0.00 (0 - 0.00) | 0.00±0.00 (0.00 - 0.01) | F=0.94 | p=0.435 |
| o: Stigonematales | 0.05±0.06 (0.01 - 0.13) | 0.00±0.00 (0 - 0.00) | 0.00±0.00 (0.00 - 0.01) | F=0.94 | p=0.435 |
| f: Hapalosiphonaceae | 0.05±0.06 (0.01 - 0.13) | 0.00±0.00 (0 - 0.00) | 0.00±0.00 (0.00 - 0.01) | F=0.94 | p=0.435 |
| g: *Mastigocoleus* | 0.05±0.06 (0.01 - 0.13) | 0.00±0.00 (0 - 0.00) | 0.00±0.00 (0.00 - 0.01) | F=0.94 | p=0.435 |
| s: *Mastigocoleus testarum* | 0.05±0.06 (0.01 - 0.13) | 0.00±0.00 (0 - 0.00) | 0.00±0.00 (0.00 - 0.01) | F=0.94 | p=0.435 |
| **p: Firmicutes** | 0.87±0.74 (0.01 - 1.35) | 15.82±22.36 (0.01 - 31.63) | 17.09±15.6 (0.11 - 35.32) | F=1.259 | p=0.341 |
| c: Bacilli | 0.87±0.74 (0.01 - 1.34) | 15.82±22.36 (0.01 - 31.63) | 17.09±15.6 (0.11 - 35.32) | F=1.259 | p=0.341 |
| o: Lactobacillales | 0.87±0.74 (0.01 - 1.34) | 15.82±22.36 (0.01 - 31.63) | 17.09±15.6 (0.11 - 35.32) | F=1.259 | p=0.341 |
| f: Enterococcaceae | 0.85±0.72 (0.01 - 1.34) | 12.54±17.72 (0.01 - 25.07) | 16.79±15.32 (0.06 - 34.96) | F=1.344 | p=0.321 |
| g: *Enterococcus* | 0.84±0.72 (0.01 - 1.32) | 12.5±17.66 (0.01 - 24.99) | 16.77±15.3 (0.06 - 34.93) | F=1.346 | p=0.32 |
| s: *Enterococcus moraviensis* | 0.83±0.71 (0.01 - 1.31) | 12.5±17.66 (0.01 - 24.99) | 16.64±15.38 (0.06 - 34.86) | F=1.318 | p=0.327 |
| s: *Enterococcus raffinosus* | 0.01±0.01 (0 - 0.01) | 0 (0) | 0.04±0.09 (0 - 0.21) | F=0.411 | p=0.678 |
| s: *Enterococcus termitis* | 0.00±0.00 (0 - 0.00) | 0.00±0.00 (0 - 0.00) | 0.08±0.11 (0 - 0.27) | F=1.084 | p=0.389 |
| g: *Vagococcus* | 0.01±0.01 (0.00 - 0.02) | 0.04±0.06 (0 - 0.08) | 0.02±0.02 (0 - 0.06) | F=0.79 | p=0.49 |
| s: *Vagococcus penaei* | 0.01±0.01 (0.00 - 0.02) | 0.04±0.06 (0 - 0.08) | 0.02±0.02 (0 - 0.06) | F=0.79 | p=0.49 |
| f: Leuconostocaceae | 0 (0) | 0 (0) | 0.00±0.01 (0 - 0.01) | F=0.438 | p=0.662 |
| g: *Leuconostoc* | 0 (0) | 0 (0) | 0.00±0.01 (0 - 0.01) | F=0.438 | p=0.662 |
| s: *Leuconostoc pseudomesenteroides*  \|*Leuconostoc pseudomesenteroides* KCTC 3652 | 0 (0) | 0 (0) | 0.00±0.01 (0 - 0.01) | F=0.438 | p=0.662 |
| f: Streptococcaceae | 0.02±0.04 (0 - 0.07) | 3.28±4.64 (0 - 6.56) | 0.3±0.39 (0.03 - 0.96) | F=2.431 | p=0.158 |
| g: *Lactococcus* | 0.02±0.04 (0 - 0.07) | 3.28±4.64 (0 - 6.56) | 0.29±0.38 (0.03 - 0.93) | F=2.438 | p=0.157 |
| s: *Lactococcus lactis*\|*Lactococcus lactis* subsp. *Lactis*  \|*Lactococcus lactis* subsp. *lactis* Il1403 | 0.02±0.04 (0 - 0.07) | 3.28±4.63 (0 - 6.55) | 0.28±0.39 (0.02 - 0.93) | F=2.438 | p=0.157 |
| s: *Lactococcus plantarum* | 0 (0) | 0.00±0.00 (0 - 0.00) | 0.01±0.01 (0 - 0.02) | F=1.887 | p=0.221 |
| g: *Streptococcus* | 0 (0) | 0 (0) | 0.00±0.01 (0 - 0.02) | F=0.438 | p=0.662 |
| s: *Streptococcus dentirousetti* | 0 (0) | 0 (0) | 0.00±0.01 (0 - 0.02) | F=0.438 | p=0.662 |
| c: Tissierellia | 0.00±0.00 (0 - 0.00) | 0.00±0.00 (0 - 0.00) | 0.00±0.00 (0 - 0.00) | F=0.055 | p=0.947 |
| o: Tissierellales | 0.00±0.00 (0 - 0.00) | 0.00±0.00 (0 - 0.00) | 0.00±0.00 (0 - 0.00) | F=0.055 | p=0.947 |
| f: Peptoniphilaceae | 0.00±0.00 (0 - 0.00) | 0.00±0.00 (0 - 0.00) | 0.00±0.00 (0 - 0.00) | F=0.055 | p=0.947 |
| g: *Anaerococcus* | 0.00±0.00 (0 - 0.00) | 0.00±0.00 (0 - 0.00) | 0.00±0.00 (0 - 0.00) | F=0.055 | p=0.947 |
| s: *Anaerococcus senegalensis*  \|*Anaerococcus senegalensis* JC48 | 0.00±0.00 (0 - 0.00) | 0.00±0.00 (0 - 0.00) | 0.00±0.00 (0 - 0.00) | F=0.055 | p=0.947 |
| **p: Proteobacteria** | 90.25±6.99 (82.19 - 94.65) | 78.84±22.71 (62.78 - 94.9) | 74.62±20.1 (57.03 - 99.31) | F=0.726 | p=0.517 |
| c: Alphaproteobacteria | 0.39±0.67 (0.00 - 1.17) | 6.12±3.95 (3.33 - 8.91) | 0.02±0.01 (0.01 - 0.04) | F=12.121 | p=0.005* |
| o: Caulobacterales | 0.00±0.00 (0 - 0.00) | 0.00±0.00 (0 - 0.00) | 0.00±0.00 (0 - 0.01) | F=0.451 | p=0.655 |
| f: Caulobacteraceae | 0.00±0.00 (0 - 0.00) | 0.00±0.00 (0 - 0.00) | 0.00±0.00 (0 - 0.01) | F=0.451 | p=0.655 |
| g: *Phenylobacterium* | 0.00±0.00 (0 - 0.00) | 0.00±0.00 (0 - 0.00) | 0.00±0.00 (0 - 0.01) | F=0.451 | p=0.655 |
| s: *Phenylobacterium koreense* | 0.00±0.00 (0 - 0.00) | 0.00±0.00 (0 - 0.00) | 0.00±0.00 (0 - 0.01) | F=0.451 | p=0.655 |
| o: Rhizobiales | 0.01±0.01 (0.00 - 0.01) | 0.01±0.01 (0 - 0.01) | 0.00±0.00 (0 - 0.01) | F=0.325 | p=0.733 |
| f: Bradyrhizobiaceae | 0.00±0.00 (0.00 - 0.00) | 0.01±0.01 (0 - 0.01) | 0.00±0.00 (0 - 0.01) | F=0.201 | p=0.823 |
| g: *Bradyrhizobium* | 0.00±0.00 (0.00 - 0.00) | 0.01±0.01 (0 - 0.01) | 0.00±0.00 (0 - 0.01) | F=0.201 | p=0.823 |
| s: *Bradyrhizobium ottawaense* | 0.00±0.00 (0.00 - 0.00) | 0.01±0.01 (0 - 0.01) | 0.00±0.00 (0 - 0.01) | F=0.201 | p=0.823 |
| f: Methylobacteriaceae | 0.00±0.01 (0 - 0.01) | 0 (0) | 0.00±0.00 (0 - 0.00) | F=1.091 | p=0.387 |
| g: *Methylobacterium* | 0.00±0.01 (0 - 0.01) | 0 (0) | 0.00±0.00 (0 - 0.00) | F=1.091 | p=0.387 |
| s: *Methylobacterium phyllostachyos* | 0.00±0.01 (0 - 0.01) | 0 (0) | 0.00±0.00 (0 - 0.00) | F=1.091 | p=0.387 |
| o: Rhodospirillales | 0.38±0.66 (0 - 1.15) | 0 (0) | 0.01±0.01 (0.01 - 0.02) | F=1.167 | p=0.365 |
| f: Acetobacteraceae | 0.38±0.66 (0 - 1.15) | 0 (0) | 0.01±0.01 (0.01 - 0.02) | F=1.167 | p=0.365 |
| g: *Asaia* | 0.38±0.66 (0 - 1.15) | 0 (0) | 0.01±0.00 (0.00 - 0.01) | F=1.167 | p=0.365 |
| s: *Asaia krungthepensis* | 0.38±0.66 (0 - 1.15) | 0 (0) | 0.01±0.00 (0.00 - 0.01) | F=1.167 | p=0.365 |
| g: *Commensalibacter* | 0 (0) | 0 (0) | 0.00±0.00 (0 - 0.01) | F=0.798 | p=0.488 |
| s: *Commensalibacter intestine*  \|*Commensalibacter intestini* A911 | 0 (0) | 0 (0) | 0.00±0.00 (0 - 0.01) | F=0.798 | p=0.488 |
| g: *Neokomagataea* | 0 (0) | 0 (0) | 0.00±0.01 (0 - 0.01) | F=0.946 | p=0.433 |
| s: *Neokomagataea tanensis*  \|*Neokomagataea tanensis* NBRC 106556 | 0 (0) | 0 (0) | 0.00±0.01 (0 - 0.01) | F=0.946 | p=0.433 |
| o: Rickettsiales | 0.00±0.00 (0 - 0.00) | 6.11±3.94 (3.33 - 8.89) | 0.00±0.00 (0 - 0.00) | F=13.487 | p=0.004* |
| f: Anaplasmataceae | 0.00±0.00 (0 - 0.00) | 6.11±3.94 (3.33 - 8.89) | 0.00±0.00 (0 - 0.00) | F=13.487 | p=0.004* |
| g: *Wolbachiae* | 0.00±0.00 (0 - 0.00) | 6.11±3.94 (3.33 - 8.89) | 0.00±0.00 (0 - 0.00) | F=13.487 | p=0.004* |
| s: *Wolbachia*\|*Wolbachia* endosymbiont of *Culex quinquefasciatus*  \|*Wolbachia* endosymbiont of *Culex quinquefasciatus* Pel | 0.00±0.00 (0 - 0.00) | 6.09±3.93 (3.31 - 8.87) | 0.00±0.00 (0 - 0.00) | F=13.441 | p=0.004* |
| s: *Wolbachia*\|*Wolbachia* sp. wRi | 0 (0) | 0.02±0.01 (0.02 - 0.02) | 0 (0) | F=56.073 | p=0* |
| o: Sphingomonadales | 0.00±0.00 (0 - 0.01) | 0 (0) | 0.00±0.00 (0 - 0.00) | F=0.771 | p=0.498 |
| f: Sphingomonadaceae | 0.00±0.00 (0 - 0.01) | 0 (0) | 0.00±0.00 (0 - 0.00) | F=0.771 | p=0.498 |
| g: *Sphingomonas* | 0.00±0.00 (0 - 0.01) | 0 (0) | 0.00±0.00 (0 - 0.00) | F=0.771 | p=0.498 |
| s: *Sphingomonas echinoides* | 0.00±0.00 (0 - 0.01) | 0 (0) | 0.00±0.00 (0 - 0.00) | F=0.771 | p=0.498 |
| c: Betaproteobacteria | 0.61±0.22 (0.4 - 0.83) | 0.33±0.08 (0.27 - 0.39) | 0.5±0.35 (0.17 - 0.91) | F=0.559 | p=0.595 |
| o: Burkholderiales | 0.61±0.22 (0.4 - 0.83) | 0.33±0.08 (0.27 - 0.39) | 0.5±0.35 (0.17 - 0.91) | F=0.559 | p=0.595 |
| f: Burkholderiaceae | 0.61±0.22 (0.4 - 0.83) | 0.33±0.08 (0.27 - 0.39) | 0.5±0.35 (0.17 - 0.91) | F=0.559 | p=0.595 |
| g: *Burkholderia* | 0.61±0.22 (0.4 - 0.83) | 0.33±0.08 (0.27 - 0.39) | 0.5±0.35 (0.17 - 0.91) | F=0.559 | p=0.595 |
| s: *Burkholderia cepacia* complex\|*Burkholderia lata* | 0.61±0.22 (0.4 - 0.83) | 0.33±0.08 (0.27 - 0.39) | 0.5±0.36 (0.17 - 0.91) | F=0.547 | p=0.602 |
| s: *pseudomallei* group\|*Burkholderia thailandensis* | 0.00±0.00 (0 - 0.00) | 0 (0) | 0.00±0.00 (0 - 0.00) | F=0.847 | p=0.468 |
| c: Deltaproteobacteria | 8.72±7.62 (0.01 - 14.18) | 12.45±17.6 (0.01 - 24.89) | 2.31±3.15 (0.01 - 6.05) | F=1.299 | p=0.331 |
| o: Desulfovibrionales | 8.72±7.62 (0.01 - 14.18) | 12.45±17.6 (0.01 - 24.89) | 2.31±3.15 (0.01 - 6.05) | F=1.299 | p=0.331 |
| f: Desulfovibrionaceae | 8.72±7.62 (0.01 - 14.18) | 12.45±17.6 (0.01 - 24.89) | 2.31±3.15 (0.01 - 6.05) | F=1.299 | p=0.331 |
| g: *Desulfovibrio* | 8.72±7.62 (0.01 - 14.18) | 12.45±17.6 (0.01 - 24.89) | 2.31±3.15 (0.01 - 6.05) | F=1.299 | p=0.331 |
| s: *Desulfovibrio arcticus* | 0.00±0.00 (0 - 0.01) | 0 (0) | 0.00±0.00 (0 - 0.01) | F=0.331 | p=0.729 |
| s: *Desulfovibrio cuneatus* | 7.36±6.82 (0.01 - 13.5) | 11.7±16.54 (0.00 - 23.4) | 1.78±2.44 (0.00 - 4.77) | F=1.402 | p=0.308 |
| s: *Desulfovibrio desulfuricans*\|*Desulfovibrio desulfuricans* subsp. *Desulfuricans*  \|*Desulfovibrio* *desulfuricans* subsp. *desulfuricans* str. ATCC 27774 | 0.06±0.06 (0 - 0.11) | 0.00±0.01 (0 - 0.01) | 0.01±0.01 (0 - 0.02) | F=2.071 | p=0.197 |
| s: *Desulfovibrio inopinatus* | 0.00±0.01 (0 - 0.01) | 0 (0) | 0.01±0.02 (0 - 0.04) | F=0.376 | p=0.7 |
| s: *Desulfovibrio intestinalis* | 0.00±0.00 (0 - 0.01) | 0.01±0.01 (0 - 0.02) | 0.00±0.00 (0 - 0.00) | F=1.514 | p=0.284 |
| s: *Desulfovibrio legallii* | 0 (0) | 0.02±0.03 (0 - 0.04) | 0.00±0.00 (0 - 0.01) | F=2.41 | p=0.16 |
| s: *Desulfovibrio litoralis* | 0.16±0.15 (0 - 0.3) | 0.11±0.16 (0 - 0.22) | 0.3±0.43 (0 - 0.96) | F=0.265 | p=0.774 |
| s: *Desulfovibrio oxamicus* | 0.94±1.34 (0 - 2.47) | 0.22±0.31 (0.00 - 0.44) | 0.15±0.28 (0 - 0.65) | F=1.102 | p=0.384 |
| s: *Desulfovibrio simplex* | 0.09±0.14 (0 - 0.26) | 0.21±0.3 (0 - 0.42) | 0.05±0.07 (0 - 0.15) | F=0.847 | p=0.468 |
| s: *Desulfovibrio vulgaris*\|*Desulfovibrio vulgaris* DP4 | 0.11±0.19 (0 - 0.33) | 0.17±0.24 (0 - 0.34) | 0.02±0.02 (0 - 0.04) | F=1.064 | p=0.395 |
| c: Gammaproteobacteria | 80.52±1.06 (79.32 - 81.34) | 59.94±36.28 (34.29 - 85.59) | 71.79±21.77 (54.57 - 98.45) | F=0.554 | p=0.598 |
| o: Aeromonadales | 0.01±0.01 (0 - 0.01) | 0.01±0.01 (0 - 0.01) | 0 (0) | F=2.4 | p=0.161 |
| f: Aeromonadaceae | 0.01±0.01 (0 - 0.01) | 0.01±0.01 (0 - 0.01) | 0 (0) | F=2.4 | p=0.161 |
| g: *Aeromonas* | 0.01±0.01 (0 - 0.01) | 0.01±0.01 (0 - 0.01) | 0 (0) | F=2.4 | p=0.161 |
| s: *Aeromonas hydrophila* | 0 (0) | 0.01±0.01 (0 - 0.01) | 0 (0) | F=2.8 | p=0.128 |
| s: *Aeromonas sobria* | 0.01±0.01 (0 - 0.01) | 0 (0) | 0 (0) | F=4.845 | p=0.048 |
| o: Chromatiales | 0.01±0.01 (0 - 0.02) | 0.02±0.03 (0 - 0.04) | 0.00±0.00 (0 - 0.00) | F=1.991 | p=0.207 |
| f: Chromatiaceae | 0.01±0.01 (0 - 0.02) | 0.01±0.01 (0 - 0.01) | 0 (0) | F=1.78 | p=0.237 |
| g: *Rhabdochromatium* | 0.01±0.01 (0 - 0.02) | 0 (0) | 0 (0) | F=2.52 | p=0.15 |
| s: *Rhabdochromatium marinum* | 0.01±0.01 (0 - 0.02) | 0 (0) | 0 (0) | F=2.52 | p=0.15 |
| g: *Thiohalocapsa* | 0 (0) | 0.01±0.01 (0 - 0.01) | 0 (0) | F=2.8 | p=0.128 |
| s: *Thiohalocapsa marina* | 0 (0) | 0.01±0.01 (0 - 0.01) | 0 (0) | F=2.8 | p=0.128 |
| f: Granulosicoccaceae | 0.00±0.00 (0 - 0.01) | 0.01±0.02 (0 - 0.03) | 0.00±0.00 (0 - 0.00) | F=2.129 | p=0.19 |
| g: *Granulosicoccus* | 0.00±0.00 (0 - 0.01) | 0.01±0.02 (0 - 0.03) | 0.00±0.00 (0 - 0.00) | F=2.129 | p=0.19 |
| s: *Granulosicoccus antarcticus*  \|*Granulosicoccus antarcticus* IMCC3135 | 0.00±0.00 (0 - 0.01) | 0.01±0.01 (0 - 0.02) | 0 (0) | F=2.134 | p=0.189 |
| s: *Granulosicoccus marinus* | 0 (0) | 0.01±0.01 (0 - 0.01) | 0.00±0.00 (0 - 0.00) | F=2.097 | p=0.193 |
| o: Enterobacteriales | 32.62±42.15 (5.21 - 81.15) | 43.17±59.95 (0.78 - 85.56) | 56.03±41.13 (0.73 - 98.42) | F=0.265 | p=0.774 |
| f: Enterobacteriaceae | 32.62±42.15 (5.21 - 81.15) | 43.17±59.95 (0.78 - 85.56) | 56.03±41.13 (0.73 - 98.42) | F=0.265 | p=0.774 |
| g: *Cedecea* | 0.00±0.00 (0 - 0.00) | 0.01±0.01 (0 - 0.02) | 0.04±0.04 (0 - 0.1) | F=1.376 | p=0.314 |
| s: *Cedecea lapagei* | 0.00±0.00 (0 - 0.00) | 0.01±0.01 (0 - 0.02) | 0.04±0.04 (0 - 0.1) | F=1.376 | p=0.314 |
| g: *Citrobacter* | 28.01±39.93 (4.16 - 74.11) | 23.26±32.55 (0.25 - 46.28) | 25.85±27.86 (0.01 - 71.87) | F=0.013 | p=0.987 |
| s: *Citrobacter freundii* complex\|*Citrobacter braakii* | 0.00±0.01 (0 - 0.01) | 0.00±0.01 (0 - 0.01) | 0.02±0.03 (0 - 0.08) | F=0.789 | p=0.491 |
| s: *Citrobacter freundii* complex\|*Citrobacter freundii* | 27.76±39.56 (4.12 - 73.43) | 23.19±32.45 (0.24 - 46.13) | 25.13±26.65 (0.01 - 68.79) | F=0.013 | p=0.987 |
| s: *Citrobacter freundii* complex\|*Citrobacter gillenii* | 0.02±0.04 (0 - 0.07) | 0.00±0.00 (0 - 0.01) | 0.00±0.00 (0 - 0.00) | F=0.966 | p=0.426 |
| s: *Citrobacter freundii* complex\|*Citrobacter murliniae* | 0.21±0.33 (0 - 0.58) | 0.06±0.08 (0.00 - 0.11) | 0.07±0.06 (0 - 0.15) | F=0.622 | p=0.564 |
| s: *Citrobacter freundii* complex\|*Citrobacter sedlakii* | 0.00±0.00 (0 - 0.00) | 0.00±0.00 (0 - 0.01) | 0.00±0.01 (0 - 0.01) | F=0.297 | p=0.752 |
| s: *Citrobacter freundii* complex\|*Citrobacter werkmanii* | 0.01±0.02 (0.00 - 0.03) | 0.00±0.00 (0 - 0.00) | 0.01±0.01 (0 - 0.02) | F=0.846 | p=0.469 |
| s: *Citrobacter koseri* | 0 (0) | 0.00±0.00 (0 - 0.00) | 0.00±0.01 (0 - 0.02) | F=0.379 | p=0.698 |
| s: *Citrobacter koseri*\|*Citrobacter koseri* ATCC BAA-895 | 0 (0) | 0.00±0.01 (0 - 0.01) | 0.61±1.27 (0 - 2.88) | F=0.502 | p=0.626 |
| g: *Cronobacter* | 0.00±0.00 (0 - 0.00) | 0.61±0.85 (0.01 - 1.21) | 0.14±0.26 (0.00 - 0.61) | F=1.684 | p=0.253 |
| s: *Cronobacter dublinensis* | 0 (0) | 0.01±0.02 (0 - 0.02) | 0.01±0.01 (0 - 0.03) | F=0.611 | p=0.569 |
| s: *Cronobacter dublinensis*\|*Cronobacter dublinensis* subsp. *lactaridi* | 0.00±0.00 (0 - 0.00) | 0.46±0.63 (0.01 - 0.91) | 0.1±0.21 (0 - 0.47) | F=1.67 | p=0.255 |
| s: *Cronobacter muytjensii*\|*Cronobacter muytjensii* ATCC 51329 | 0 (0) | 0 (0) | 0.00±0.01 (0 - 0.02) | F=0.438 | p=0.662 |
| s: *Cronobacter sakazakii*\|*Cronobacter sakazakii* ATCC BAA-894 | 0 (0) | 0.13±0.19 (0 - 0.26) | 0.02±0.04 (0 - 0.1) | F=1.934 | p=0.215 |
| s: *Cronobacter turicensis*\|*Cronobacter turicensis* z3032 | 0 (0) | 0.00±0.01 (0 - 0.01) | 0.00±0.00 (0 - 0.01) | F=1.454 | p=0.296 |
| g: *Enterobacter* | 0.4±0.18 (0.25 - 0.6) | 0.43±0.6 (0.01 - 0.85) | 0.54±0.41 (0.05 - 1.14) | F=0.125 | p=0.885 |
| s: *Enterobacter aerogenes*\|*Enterobacter aerogenes* KCTC 2190 | 0.02±0.00 (0.02 - 0.02) | 0.13±0.19 (0.00 - 0.26) | 0.06±0.06 (0.00 - 0.17) | F=1.037 | p=0.403 |
| s: *Enterobacter cloacae* complex  \|*Enterobacter asburiae*\|*Enterobacter* *asburiae* LF7a | 0.04±0.03 (0.00 - 0.07) | 0.13±0.19 (0 - 0.26) | 0.09±0.06 (0.00 - 0.17) | F=0.766 | p=0.5 |
| s: *Enterobacter cloacae* complex\|*Enterobacter cloacae* | 0.31±0.22 (0.12 - 0.55) | 0.15±0.2 (0.01 - 0.29) | 0.37±0.36 (0.05 - 0.97) | F=0.37 | p=0.704 |
| s: *Enterobacter cloacae* complex  \|*Enterobacter cloacae*\|*Enterobacter cloacae* subsp. *dissolvens* | 0.01±0.02 (0 - 0.03) | 0.01±0.01 (0 - 0.02) | 0.01±0.01 (0 - 0.03) | F=0.098 | p=0.908 |
| s: *Enterobacter* sp. 638 | 0.03±0.04 (0 - 0.07) | 0.01±0.01 (0 - 0.02) | 0.01±0.01 (0.00 - 0.03) | F=0.291 | p=0.756 |
| g: *Erwinia* | 0.46±0.73 (0.00 - 1.3) | 0.05±0.06 (0.00 - 0.09) | 0.02±0.02 (0.00 - 0.04) | F=1.281 | p=0.336 |
| s: *Erwinia aphidicola* | 0.01±0.01 (0 - 0.02) | 0 (0) | 0.00±0.00 (0 - 0.00) | F=1.133 | p=0.375 |
| s: *Erwinia persicina* | 0.02±0.03 (0 - 0.05) | 0.02±0.03 (0.00 - 0.04) | 0.00±0.00 (0 - 0.00) | F=1.108 | p=0.382 |
| s: *Erwinia psidii* | 0 (0) | 0.01±0.02 (0 - 0.03) | 0.00±0.00 (0 - 0.01) | F=1.96 | p=0.211 |
| s: *Erwinia pyrifoliae*\|*Erwinia pyrifoliae* Ep1/96 | 0.44±0.75 (0.00 - 1.3) | 0.01±0.02 (0 - 0.02) | 0.02±0.02 (0.00 - 0.04) | F=1.157 | p=0.368 |
| g: *Escherichia* | 0.00±0.00 (0 - 0.00) | 0.01±0.01 (0 - 0.01) | 0.22±0.31 (0 - 0.76) | F=1.017 | p=0.41 |
| s: *Escherichia fergusonii*\|*Escherichia fergusonii* ATCC 35469 | 0.00±0.00 (0 - 0.00) | 0.01±0.01 (0 - 0.01) | 0.22±0.31 (0 - 0.76) | F=1.017 | p=0.41 |
| g: *Gibbsiella* | 0.00±0.00 (0 - 0.00) | 0.01±0.01 (0 - 0.02) | 0.00±0.01 (0 - 0.01) | F=0.939 | p=0.435 |
| s: *Gibbsiella greigii* | 0.00±0.00 (0 - 0.00) | 0.01±0.01 (0 - 0.02) | 0.00±0.01 (0 - 0.01) | F=0.939 | p=0.435 |
| g: *Hafnia* | 0.00±0.00 (0 - 0.00) | 0.02±0.03 (0 - 0.04) | 0.01±0.02 (0 - 0.05) | F=0.679 | p=0.538 |
| s: *Hafnia alvei* | 0 (0) | 0 (0) | 0.01±0.01 (0 - 0.03) | F=0.438 | p=0.662 |
| s: *Hafnia paralvei* | 0.00±0.00 (0 - 0.00) | 0.02±0.03 (0 - 0.04) | 0.00±0.01 (0 - 0.02) | F=1.635 | p=0.262 |
| g: *Klebsiella* | 2.39±2.31 (0.07 - 4.68) | 9.08±12.36 (0.34 - 17.83) | 26.53±35.01 (0.58 - 87.25) | F=0.833 | p=0.474 |
| s: *Klebsiella oxytoca* | 2.28±2.29 (0.04 - 4.62) | 8.17±11.08 (0.33 - 16.01) | 25.75±34.75 (0.52 - 86.25) | F=0.815 | p=0.481 |
| s: *Klebsiella oxytoca*\|*Klebsiella oxytoca* KCTC 1686 | 0.00±0.00 (0 - 0.01) | 0.03±0.04 (0 - 0.05) | 0.06±0.08 (0.00 - 0.2) | F=0.879 | p=0.457 |
| s: *Klebsiella pneumoniae*\|*Klebsiella pneumoniae* subsp. *Pneumoniae*  \|*Klebsiella pneumoniae* subsp. *pneumoniae* MGH 78578 | 0.01±0.00 (0.00 - 0.01) | 0.25±0.35 (0.00 - 0.49) | 0.22±0.19 (0.05 - 0.45) | F=1.444 | p=0.299 |
| s: *Klebsiella quasipneumoniae*\|*Klebsiella quasipneumoniae*  subsp. *quasipneumoniae* | 0.00±0.00 (0 - 0.00) | 0.03±0.04 (0.00 - 0.06) | 0.06±0.03 (0.01 - 0.09) | F=3.172 | p=0.105 |
| s: *Klebsiella* *quasipneumoniae*\|*Klebsiella* *quasipneumoniae*  subsp. *similipneumoniae* | 0.09±0.12 (0.02 - 0.23) | 0.03±0.05 (0.00 - 0.07) | 0.06±0.05 (0 - 0.14) | F=0.394 | p=0.689 |
| s: *Klebsiella variicola* | 0.01±0.01 (0 - 0.02) | 0.57±0.8 (0.00 - 1.14) | 0.37±0.33 (0.01 - 0.69) | F=1.379 | p=0.313 |
| s: *Klebsiella variicola*\|*Klebsiella variicola* At-22 | 0.00±0.00 (0 - 0.00) | 0.00±0.00 (0.00 - 0.01) | 0.01±0.01 (0 - 0.02) | F=1.371 | p=0.315 |
| g: *Kluyvera* | 0.07±0.07 (0.02 - 0.15) | 0.22±0.28 (0.02 - 0.42) | 0.3±0.21 (0 - 0.56) | F=1.198 | p=0.357 |
| s: *Kluyvera cryocrescens* | 0.07±0.07 (0.02 - 0.15) | 0.21±0.27 (0.02 - 0.4) | 0.28±0.21 (0 - 0.56) | F=1.119 | p=0.379 |
| s: *Kluyvera intermedia* | 0 (0) | 0.01±0.01 (0 - 0.02) | 0.01±0.02 (0 - 0.05) | F=0.498 | p=0.628 |
| g: *Kosakonia* | 0.00±0.00 (0 - 0.01) | 0 (0) | 0.00±0.00 (0 - 0.01) | F=0.382 | p=0.696 |
| s: *Kosakonia sacchari* | 0.00±0.00 (0 - 0.01) | 0 (0) | 0.00±0.00 (0 - 0.01) | F=0.382 | p=0.696 |
| g: *Leclercia* | 0.00±0.00 (0.00 - 0.01) | 0.03±0.04 (0.01 - 0.06) | 0.05±0.05 (0 - 0.12) | F=1.307 | p=0.329 |
| s: *Leclercia adecarboxylata* | 0.00±0.00 (0.00 - 0.01) | 0.03±0.04 (0.01 - 0.06) | 0.05±0.05 (0 - 0.12) | F=1.307 | p=0.329 |
| g: *Mangrovibacter* | 0.13±0.12 (0 - 0.24) | 0.01±0.01 (0 - 0.01) | 0.01±0.01 (0 - 0.03) | F=3.714 | p=0.08 |
| s: *Mangrovibacter* *plantisponsor* | 0.13±0.12 (0 - 0.24) | 0.01±0.01 (0 - 0.01) | 0.01±0.01 (0 - 0.03) | F=3.714 | p=0.08 |
| g: *Morganella* | 0 (0) | 0.63±0.88 (0 - 1.25) | 0.01±0.02 (0 - 0.04) | F=2.748 | p=0.132 |
| s: *Morganella morganii*\|*Morganella morganii* subsp. *Morganii*  \|*Morganella morganii* subsp. *morganii* KT | 0 (0) | 0.63±0.88 (0 - 1.25) | 0.01±0.02 (0 - 0.04) | F=2.748 | p=0.132 |
| g: *Pantoea* | 0 (0) | 0.03±0.04 (0 - 0.06) | 0.01±0.01 (0 - 0.01) | F=1.931 | p=0.215 |
| s: *Pantoea dispersa* | 0 (0) | 0.00±0.00 (0 - 0.00) | 0.01±0.01 (0 - 0.01) | F=1.569 | p=0.274 |
| s: Pantoea eucalypti | 0 (0) | 0.01±0.01 (0 - 0.01) | 0.00±0.00 (0 - 0.00) | F=2.593 | p=0.144 |
| s: *Pantoea stewartii*\|*Pantoea stewartii* subsp. *indologenes* | 0 (0) | 0.01±0.01 (0 - 0.01) | 0.00±0.00 (0 - 0.00) | F=2.318 | p=0.169 |
| s: *Pantoea wallisii* | 0 (0) | 0.01±0.02 (0 - 0.03) | 0.00±0.00 (0 - 0.00) | F=2.515 | p=0.15 |
| g: *Pectobacterium* | 0 (0) | 0.00±0.00 (0 - 0.00) | 0.02±0.03 (0 - 0.07) | F=0.655 | p=0.549 |
| s: *Pectobacterium atrosepticum*  \|*Pectobacterium* *atrosepticum* SCRI1043 | 0 (0) | 0.00±0.00 (0 - 0.00) | 0.01±0.03 (0 - 0.07) | F=0.416 | p=0.675 |
| s: *Pectobacterium carotovorum*  \|*Pectobacterium carotovorum* subsp. *actinidiae* | 0 (0) | 0 (0) | 0.00±0.01 (0 - 0.01) | F=0.438 | p=0.662 |
| g: *Providencia* | 0.06±0.1 (0 - 0.17) | 0.04±0.06 (0.00 - 0.08) | 0.1±0.21 (0 - 0.47) | F=0.095 | p=0.911 |
| s: *Providencia alcalifaciens*\|*Providencia alcalifaciens* DSM 30120 | 0.05±0.08 (0 - 0.14) | 0.04±0.06 (0.00 - 0.08) | 0.1±0.21 (0 - 0.47) | F=0.124 | p=0.885 |
| s: *Providencia stuartii*\|*Providencia stuartii* MRSN 2154 | 0.01±0.02 (0 - 0.04) | 0 (0) | 0 (0) | F=1.225 | p=0.35 |
| g: *Pseudocitrobacter* | 0 (0) | 0.02±0.03 (0 - 0.05) | 0.00±0.01 (0 - 0.02) | F=1.871 | p=0.223 |
| s: *Pseudocitrobacter anthropi* | 0 (0) | 0.02±0.03 (0 - 0.05) | 0.00±0.01 (0 - 0.02) | F=1.871 | p=0.223 |
| g: *Raoultella* | 0.03±0.03 (0.00 - 0.05) | 8.68±12.12 (0.11 - 17.25) | 2.16±3.87 (0.00 - 9.06) | F=1.594 | p=0.269 |
| s: *Raoultella electrica* | 0 (0) | 0.02±0.03 (0 - 0.04) | 0.01±0.01 (0 - 0.03) | F=1.1 | p=0.384 |
| s: *Raoultella ornithinolytica* | 0.00±0.00 (0 - 0.00) | 0 (0) | 0.00±0.01 (0 - 0.02) | F=0.274 | p=0.768 |
| s: *Raoultella ornithinolytica*\|*Raoultella ornithinolytica* B6 | 0 (0) | 0 (0) | 0.00±0.01 (0 - 0.02) | F=0.438 | p=0.662 |
| s: *Raoultella planticola*\|*Raoultella planticola* ATCC 33531 | 0.00±0.00 (0.00 - 0.00) | 8.63±12.05 (0.11 - 17.15) | 2.12±3.86 (0.00 - 9.01) | F=1.602 | p=0.267 |
| s: *Raoultella terrigena* | 0.03±0.03 (0 - 0.05) | 0.03±0.05 (0 - 0.06) | 0.03±0.03 (0 - 0.07) | F=0.037 | p=0.964 |
| g: *Rosenbergiella* | 0.01±0.01 (0 - 0.01) | 0.00±0.00 (0 - 0.01) | 0 (0) | F=2.671 | p=0.137 |
| s: *Rosenbergiella australoborealis* | 0.01±0.01 (0 - 0.01) | 0.00±0.00 (0 - 0.01) | 0 (0) | F=2.671 | p=0.137 |
| g: *Salmonella* | 0.05±0.08 (0 - 0.14) | 0.00±0.00 (0 - 0.00) | 0.01±0.02 (0 - 0.06) | F=0.72 | p=0.519 |
| s: *Salmonella enterica*\|*Salmonella enterica* subsp. arizonae | 0 (0) | 0 (0) | 0.00±0.01 (0 - 0.02) | F=0.438 | p=0.662 |
| s: *Salmonella enterica*\|*Salmonella enterica* subsp. *Enterica*  \|*Salmonella enterica* subsp. *enterica* serovar Typhimurium | 0.00±0.00 (0 - 0.00) | 0.00±0.00 (0 - 0.00) | 0.01±0.02 (0 - 0.04) | F=0.555 | p=0.597 |
| s: *Salmonella subterranea* | 0.05±0.08 (0 - 0.14) | 0 (0) | 0 (0) | F=1.225 | p=0.35 |
| g: *Siccibacter* | 0.97±1.64 (0.02 - 2.87) | 0.02±0.02 (0.01 - 0.04) | 0.01±0.01 (0.00 - 0.03) | F=1.235 | p=0.347 |
| s: *Siccibacter colletis* | 0.01±0.01 (0 - 0.01) | 0 (0) | 0 (0) | F=3.86 | p=0.074 |
| s: *Siccibacter turicensis* | 0.01±0.01 (0 - 0.01) | 0.00±0.01 (0 - 0.01) | 0.00±0.00 (0 - 0.01) | F=0.211 | p=0.815 |
| s: *Siccibacter turicensis*\|*Siccibacter turicensis* LMG 23730 | 0.96±1.64 (0.01 - 2.85) | 0.02±0.03 (0 - 0.04) | 0.01±0.01 (0.00 - 0.03) | F=1.215 | p=0.352 |
| g: *Trabulsiella* | 0.03±0.03 (0 - 0.06) | 0.00±0.00 (0 - 0.01) | 0.01±0.01 (0 - 0.03) | F=1.834 | p=0.229 |
| s: *Trabulsiella guamensis* | 0.03±0.03 (0 - 0.06) | 0.00±0.00 (0 - 0.01) | 0.01±0.01 (0 - 0.03) | F=1.834 | p=0.229 |
| o: Oceanospirillales | 0.00±0.00 (0 - 0.00) | 0.00±0.01 (0 - 0.01) | 0.01±0.01 (0 - 0.03) | F=0.704 | p=0.527 |
| f: Halomonadaceae | 0.00±0.00 (0 - 0.00) | 0.00±0.01 (0 - 0.01) | 0.01±0.01 (0 - 0.03) | F=0.704 | p=0.527 |
| g: *Halomonas* | 0.00±0.00 (0 - 0.00) | 0.00±0.01 (0 - 0.01) | 0.01±0.01 (0 - 0.03) | F=0.704 | p=0.527 |
| s: *Halomonas stevensii*\|*Halomonas stevensii* S18214 | 0.00±0.00 (0 - 0.00) | 0.00±0.01 (0 - 0.01) | 0.01±0.01 (0 - 0.03) | F=0.704 | p=0.527 |
| o: Orbales | 47.87±41.37 (0.18 - 74.08) | 16.74±23.66 (0.01 - 33.47) | 15.74±24.56 (0.01 - 56.05) | F=1.166 | p=0.366 |
| f: Orbaceae | 47.87±41.37 (0.18 - 74.08) | 16.74±23.66 (0.01 - 33.47) | 15.74±24.56 (0.01 - 56.05) | F=1.166 | p=0.366 |
| g: *Gilliamella* | 2.28±2.53 (0.01 - 5.02) | 0.3±0.43 (0.00 - 0.61) | 0.18±0.38 (0 - 0.86) | F=2.32 | p=0.169 |
| s: *Gilliamella apicola* | 2.28±2.53 (0.01 - 5.02) | 0.3±0.43 (0.00 - 0.61) | 0.18±0.38 (0 - 0.86) | F=2.32 | p=0.169 |
| g: *Orbus* | 45.59±39.35 (0.17 - 69.06) | 16.43±23.23 (0.01 - 32.86) | 15.56±24.21 (0.01 - 55.19) | F=1.09 | p=0.387 |
| s: *Orbus hercynius* | 0.00±0.00 (0 - 0.01) | 16.02±22.66 (0.00 - 32.04) | 4.55±10 (0.00 - 22.44) | F=1.213 | p=0.353 |
| s: *Orbus sasakiae* | 45.59±39.34 (0.17 - 69.06) | 0.41±0.57 (0.01 - 0.81) | 11.01±24.53 (0.01 - 54.9) | F=1.991 | p=0.207 |
| o: Pseudomonadales | 0.01±0.01 (0 - 0.03) | 0 (0) | 0.01±0.01 (0 - 0.02) | F=0.831 | p=0.474 |
| f: Pseudomonadaceae | 0.01±0.01 (0 - 0.03) | 0 (0) | 0.01±0.01 (0 - 0.02) | F=0.831 | p=0.474 |
| g: *Pseudomonas* | 0.00±0.01 (0 - 0.01) | 0 (0) | 0.01±0.01 (0 - 0.02) | F=0.831 | p=0.474 |
| s: *Pseudomonas aeruginosa* group\|*Pseudomonas aeruginosa* | 0.00±0.00 (0 - 0.00) | 0 (0) | 0.00±0.00 (0 - 0.01) | F=0.631 | p=0.56 |
| s: *Pseudomonas composti* | 0 (0) | 0 (0) | 0.00±0.01 (0 - 0.01) | F=0.998 | p=0.416 |
| s: *Pseudomonas graminis* | 0.00±0.01 (0 - 0.01) | 0 (0) | 0.00±0.00 (0 - 0.00) | F=0.942 | p=0.434 |
| s: *Pseudomonas knackmussii*\|*Pseudomonas knackmussii* B13 | 0 (0) | 0 (0) | 0.00±0.01 (0 - 0.02) | F=0.438 | p=0.662 |
| g: *Serpens* | 0.01±0.01 (0 - 0.02) | 0 (0) | 0 (0) | F=1.225 | p=0.35 |
| s: *Serpens flexibilis* | 0.01±0.01 (0 - 0.02) | 0 (0) | 0 (0) | F=1.225 | p=0.35 |
| o: *Thiotrichales* | 0.00±0.01 (0 - 0.01) | 0 (0) | 0.00±0.00 (0 - 0.00) | F=1.042 | p=0.402 |
| f: *Thiotrichaceae* | 0.00±0.01 (0 - 0.01) | 0 (0) | 0.00±0.00 (0 - 0.00) | F=1.042 | p=0.402 |
| g: *Beggiatoa* | 0.00±0.01 (0 - 0.01) | 0 (0) | 0.00±0.00 (0 - 0.00) | F=1.042 | p=0.402 |
| s: *Beggiatoa* *alba*\|*Beggiatoa* *alba* B18LD | 0.00±0.01 (0 - 0.01) | 0 (0) | 0.00±0.00 (0 - 0.00) | F=1.042 | p=0.402 |
| Unassigned | 0.24±0.16 (0.08 - 0.4) | 0.15±0.15 (0.05 - 0.26) | 0.25±0.12 (0.06 - 0.36) | F=0.391 | p=0.69 |

**Supplementary Table S4** Bacterial genera detected in the core phyla of adult male flies of *Bactrocera melastomatos* and *Bactrocera umbrosa* from Peninsular Malaysia; +, present; –, absent

| **Phylum**/Genus | *B. melastomatos*  (Awana) | *B. melastomatos*  (U. Malaya) | *B. umbrosa*  (U. Malaya) |
| --- | --- | --- | --- |
| ***Actinobacteria***  *Corynebacterium*  *Mycobacterium* | **–**  **+** | **–**  **–** | **+**  **–** |
| *Williamsia* | **+** | **–** | **–** |
| *Leifsonia* | **+** | + | **+** |
| *Propionobacterium* | + | + | + |
| ***Armatimonadetes***  *Chthonomonas* | + | **–** | **–** |
| ***Bacteroidetes***  *Bacteroides* | + | + | + |
| *Dysgonomonas* | **+** | + | **+** |
| *Microbacter* | **+** | + | **+** |
| *Parabacteroides* | **+** | + | **–** |
| *Porphyromonas* | **+** | + | **+** |
| *Chishuiella* | **+** | + | **+** |
| *Chryseobacterium* | **+** | **–** | **–** |
| *Flavobacterium* | + | + | + |
| ***Cyanobacteria***  *Mastigocoleus* | **+** | **+** | + |
| ***Firmicutes***  *Enterococcus* | + | **+** | **+** |
| *Vagococcus*  *Leuconostoc* | +  **–** | +  **–** | +  + |
| *Lactococcus*  *Streptococcus*  *Anerococcus* | **+**  **–**  **+** | **+**  **–**  **+** | +  +  + |
| ***Proteobacteria***  *Phenylobacterium*  *Bradyrhizobium* | +  + | +  + | +  + |
| *Methylobacterium*  *Asaia*  *Commensalibacter*  *Neokomagataea*  *Wolbachleae* | +  +  **–**  **–**  **+** | **–**  **–**  **–**  **–**  **+** | +  +  +  +  + |
| *Sphingomonas* | + | **–** | + |
| *Burkholderia* | + | + | + |
| *Desulfovibrio* | **+** | **+** | + |
| *Aeromonas* | **+** | **+** | **–** |
| *Rhabdochromatium*  *Thiohalocapsa* | **+**  **–** | **–**  **+** | **–**  **–** |
| *Granulosicoccus* | **+** | + | **+** |
| *Cedecia* | + | + | **+** |
| *Citrobacter* | **+** | + | **+** |
| *Cronobacter* | **+** | + | **+** |
| *Enterobacter*  *Erwinia*  *Escherichia*  *Gibbsiella*  *Hafnia* | +  +  +  +  + | +  +  +  +  + | +  +  +  +  + |
| *Klebsiella* | + | + | + |
| *Kluyvera* | + | **+** | + |
| *Kosakonia* | + | **–** | + |
| *Leclercia* | + | **+** | + |
| *Mangrovibacter*  *Morganella*  *Pantoea*  *Pectobacterium* | +  **–**  **–**  **–** | **+**  **+**  **+**  **+** | +  +  +  + |
| *Providencia* | + | + | + |
| *Pseudocitrobacter* | **–** | + | + |
| *Raoultella* | + | + | + |
| *Rosenbergiella* | + | **+** | **–** |
| *Salmonella* | + | + | + |
| *Siccibacter* | + | **+** | + |
| *Trabulsiella* | + | **+** | + |
| *Halomonas* | + | **+** | + |
| *Gilliamella* | + | **+** | **+** |
| *Orbus* | **+** | **+** | **+** |
| *Pseudomonas* | + | **–** | **+** |
| *Serpens* | **+** | **–** | **–** |
| *Beggiatoa* | **+** | **–** | **+** |
| Total (64) | 54 | 47 | 54 |
